# Connecting the multiple dimensions of global soil fungal diversity

**DOI:** 10.1126/sciadv.adj8016

**Published:** 2023-11-29

**Authors:** Vladimir Mikryukov, Olesya Dulya, Alexander Zizka, Mohammad Bahram, Niloufar Hagh-Doust, Sten Anslan, Oleh Prylutskyi, Manuel Delgado-Baquerizo, Fernando T. Maestre, Henrik Nilsson, Jaan Pärn, Maarja Öpik, Mari Moora, Martin Zobel, Mikk Espenberg, Ülo Mander, Abdul Nasir Khalid, Adriana Corrales, Ahto Agan, Aída-M. Vasco-Palacios, Alessandro Saitta, Andrea Rinaldi, Annemieke Verbeken, Bobby Sulistyo, Boris Tamgnoue, Brendan Furneaux, Camila Duarte Ritter, Casper Nyamukondiwa, Cathy Sharp, César Marín, Daniyal Gohar, Darta Klavina, Dipon Sharmah, Dong-Qin Dai, Eduardo Nouhra, Elisabeth Machteld Biersma, Elisabeth Rähn, Erin Cameron, Eske De Crop, Eveli Otsing, Evgeny Davydov, Felipe Albornoz, Francis Brearley, Franz Buegger, Geoffrey Zahn, Gregory Bonito, Inga Hiiesalu, Isabel Barrio, Jacob Heilmann-Clausen, Jelena Ankuda, Jiri Doležal, John Kupagme, Jose Maciá-Vicente, Joseph Djeugap Fovo, József Geml, Juha Alatalo, Julieta Alvarez-Manjarrez, Kadri Põldmaa, Kadri Runnel, Kalev Adamson, Kari-Anne Bråthen, Karin Pritsch, Kassim Tchan Issifou, Kęstutis Armolaitis, Kevin Hyde, Kevin K. Newsham, Kristel Panksep, Adebola Azeez Lateef, Linda Hansson, Louis Lamit, Malka Saba, Maria Tuomi, Marieka Gryzenhout, Marijn Bauters, Meike Piepenbring, Nalin N. Wijayawardene, Nourou Yorou, Olavi Kurina, Peter Mortimer, Peter Meidl, Petr Kohout, Rasmus Puusepp, Rein Drenkhan, Roberto Garibay-Orijel, Roberto Godoy, Saad Alkahtani, Saleh Rahimlou, Sergey Dudov, Sergei Põlme, Soumya Ghosh, Sunil Mundra, Talaat Ahmed, Tarquin Netherway, Terry Henkel, Tomas Roslin, Vincent Nteziryayo, Vladimir Fedosov, Vladimir Onipchenko, Weeragalle Arachchillage Erandi Yasanthika, Young Lim, Michael Van Nuland, Nadejda Soudzilovskaia, Alexandre Antonelli, Urmas Kõljalg, Kessy Abarenkov, Leho Tedersoo

**Affiliations:** ^1^ Institute of Ecology and Earth Sciences, University of Tartu, Tartu 50409, Estonia.; ^2^ Department of Biology, Philipps-University, Marburg 35032, Germany.; ^3^ Department of Ecology, Swedish University of Agricultural Sciences, Uppsala 75007, Sweden.; ^4^ Department of Mycology and Plant Resistance, School of Biology, V.N. Karazin Kharkiv National University, Kharkiv 61022, Ukraine.; ^5^ Laboratorio de Biodiversidad y Funcionamiento Ecosistemico, Instituto de Recursos Naturales y Agrobiología de Sevilla (IRNAS), Consejo Superior de Investigaciones Científicas, Sevilla 41012, Spain.; ^6^ Instituto Multidisciplinar para el Estudio del Medio ‘Ramón Margalef’ and Departamento de Ecología, Universidad de Alicante, Alicante 03690, Spain.; ^7^ Gothenburg Global Biodiversity Centre, University of Gothenburg, Gothenburg 40530, Sweden.; ^8^ Institute of Botany, University of the Punjab, Pakistan 54590, Pakistan.; ^9^ Centro de Investigaciones en Microbiología y Biotecnología-UR (CIMBIUR), Universidad del Rosario, Bogotá 111221, Colombia.; ^10^ Institute of Forestry and Engineering, Estonian University of Life Sciences, Tartu 51006, Estonia.; ^11^ Grupo de BioMicro y Microbiología Ambiental, Escuela de Microbiologia, Universidad de Antioquia UdeA, Medellin 050010, Colombia.; ^12^ Department of Agricultural, Food and Forest Sciences, University of Palermo, Palermo 90128, Italy.; ^13^ Department of Biomedical Sciences, University of Cagliari, Cagliari 09124, Italy.; ^14^ Department Biology, Ghent University, Ghent 9000, Belgium.; ^15^ Department of Crop Science, University of Dschang, Dschang, Cameroon.; ^16^ Department of Biological and Environmental Science, University of Jyväskylä, Jyväskylä 40014, Finland.; ^17^ Instituto Juruá, Manaus 69083, Brazil.; ^18^ Department of Biological Sciences and Biotechnology, Botswana International University of Science and Technology, Palapye 10071, Botswana.; ^19^ Natural History Museum of Zimbabwe, Bulawayo, Zimbabwe.; ^20^ Centro de Investigación e Innovación para el Cambio Climático (CiiCC), Universidad SantoTomás, Valdivia, Chile.; ^21^ Center of Mycology and Microbiology, University of Tartu, Tartu 50409, Estonia.; ^22^ Latvian State Forest Research Institute Silava, Salaspils 2169, Latvia.; ^23^ Department of Botany, Jawaharlal Nehru Rajkeeya Mahavidyalaya, Pondicherry University, Port Blair 744101, India.; ^24^ College of Biological Resource and Food Engineering, Qujing Normal University, Qujing, Yunnan 655011, China.; ^25^ Instituto Multidisciplinario de Biología Vegetal (CONICET), Universidad Nacional de Córdoba, Cordoba 5000, Argentina.; ^26^ Natural History Museum of Denmark, Copenhagen 1123, Denmark.; ^27^ British Antarctic Survey, NERC, High Cross, Cambridge CB3 0ET, UK.; ^28^ Department of Environmental Science, Saint Mary's University, Halifax B3H 3C3, Canada.; ^29^ Altai State University, Barnaul 656049, Russia.; ^30^ Land and Water, Commonwealth Scientific and Industrial Research Organisation (CSIRO), Wembley 6014, Australia.; ^31^ Department of Natural Sciences, Manchester Metropolitan University, Manchester M1 5GD, UK.; ^32^ Helmholtz Zentrum München, Neuherberg 85764, Germany.; ^33^ Biology Department, Utah Valley University, Orem, UT 84058, USA.; ^34^ Plant, Soil and Microbial Sciences, Michigan State University, East Lansing, MI 48824-6254, USA.; ^35^ Faculty of Natural and Environmental Sciences, Agricultural University of Iceland, Reykjavík 112, Iceland.; ^36^ Center for Macroecology, Evolution and Climate, University of Copenhagen, Copenhagen 1350, Denmark.; ^37^ Vokė branch, Institute of Agriculture, Lithuanian Research Centre for Agriculture and Forestry (LAMMC), Vilnius LT-02232, Lithuania.; ^38^ Department of Botany, Faculty of Science, University of South Bohemia, České Budějovice 37005, Czech Republic.; ^39^ Department of Environmental Sciences, Plant Ecology and Nature Conservation, Wageningen University and Research, Wageningen 6708, Netherlands.; ^40^ ELKH-EKKE Lendület Environmental Microbiome Research Group, Eszterházy Károly Catholic University, Eger 3300, Hungary.; ^41^ Environmental Science Center, Qatar University, Doha, Qatar.; ^42^ Instituto de Biología, Universidad Nacional Autónoma de México, Ciudad de México 04510, Mexico.; ^43^ Natural History Museum, University of Tartu, Tartu 51003, Estonia.; ^44^ Department of Arctic and Marine Biology, The Arctic University of Norway, Tromsø 9019, Norway.; ^45^ Research Unit Tropical Mycology and Plants-Soil Fungi Interactions, University of Parakou, Parakou 00229, Benin.; ^46^ Department of Silviculture and Ecology, Institute of Forestry, Lithuanian Research Centre for Agriculture and Forestry (LAMMC), Girionys 53101, Lithuania.; ^47^ Center of Excellence in Fungal Research, Mae Fah Luang University, Chiang Rai 57100, Thailand.; ^48^ Chair of Hydrobiology and Fishery, Estonian University of Life Sciences, Tartu 51006, Estonia.; ^49^ Department of Plant Biology, Faculty of Life Science, University of Ilorin, Ilorin 240102, Nigeria.; ^50^ Department of Forest Sciences, University of Helsinki, Helsinki 00014, Finland.; ^51^ Gothenburg Centre for Sustainable Development, Gothenburg 41133, Sweden.; ^52^ Department of Biology, Syracuse University, Syracuse 13244, USA.; ^53^ Department of Plant Sciences, Quaid-i-Azam University, Islamabad 45320, Pakistan.; ^54^ Department of Genetics, Faculty of Natural and Agricultural Sciences, University of the Free State, Bloemfontein 9300, South Africa.; ^55^ Department of Environment, Faculty of Bioscience Engineering, Ghent University, Ghent 9000, Belgium.; ^56^ Mycology Working Group, Goethe University Frankfurt am Main, Frankfurt am Main 60438, Germany.; ^57^ College of Biological Resource and Food Engineering, Qujing Normal University, Qujing, China.; ^58^ Institute of Agricultural and Environmental Sciences, Estonian University of Life Sciences, Tartu 51006, Estonia.; ^59^ Center For Mountain Futures, Kunming Institute of Botany, Chinese Academy of Sciences, Kunming 650201, China.; ^60^ Freie Universität Berlin, Institut für Biologie, Berlin 14195, Germany.; ^61^ Institute of Microbiology, Czech Academy of Sciences, Prague, Czech Republic.; ^62^ Instituto Ciencias Ambientales y Evolutivas, Universidad Austral de Chile, Valdivia, Chile.; ^63^ Department of Zoology, College of Science, King Saud University, Riyadh 11451, Saudi Arabia.; ^64^ Department of Ecology and Plant Geography, Moscow Lomonosov State University, Moscow 119234, Russia.; ^65^ Department of Biology, College of Science, United Arab Emirates University (UAEU), Al Ain, UAE.; ^66^ Department of Biological Sciences, California State Polytechnic University, Arcata, CA 95521, USA.; ^67^ Department of Food Science and Technology, University of Burundi, Bujumbura Burundi.; ^68^ School of Biological Sciences and Institute of Microbiology, Seoul National University, Seoul 08826, Korea.; ^69^ Society for the Protection of Underground Networks (SPUN), Dover, DE 19901, USA.; ^70^ Centre for Environmental Sciences, Hasselt University, Hasselt 3500, Belgium.; ^71^ Royal Botanic Gardens, Kew, Richmond TW9 3AE, UK.

## Abstract

How the multiple facets of soil fungal diversity vary worldwide remains virtually unknown, hindering the management of this essential species-rich group. By sequencing high-resolution DNA markers in over 4000 topsoil samples from natural and human-altered ecosystems across all continents, we illustrate the distributions and drivers of different levels of taxonomic and phylogenetic diversity of fungi and their ecological groups. We show the impact of precipitation and temperature interactions on local fungal species richness (alpha diversity) across different climates. Our findings reveal how temperature drives fungal compositional turnover (beta diversity) and phylogenetic diversity, linking them with regional species richness (gamma diversity). We integrate fungi into the principles of global biodiversity distribution and present detailed maps for biodiversity conservation and modeling of global ecological processes.

## Teaser

Water–energy balance defines fungal alpha diversity, while energy variability links phylogenetic, beta, and gamma diversity.

## INTRODUCTION

As the largest contributor to global biomass after plants and bacteria (*1*), soil-inhabiting fungi play crucial roles in maintaining the health, productivity, and nutrient cycling of terrestrial ecosystems (*2*–*4*). Yet, our understanding of fungal diversity distribution across the globe is incomplete, hampering analysis and prediction of diversity-functioning relationships in changing environments. The relatively recent emergence of high-throughput molecular identification offers the tools for large-scale surveys of diverse fungal communities (*5*–*11*). Pioneering global studies, incorporating hundreds of samples and focusing on dominant phylotypes (*5*, *7*, *9*, *12*), have consistently reported a major role of precipitation in soil fungal alpha diversity (i.e., plot-scale diversity) and the existence of a latitudinal diversity gradient (the pattern of poleward declines of biodiversity intrinsic to many large groups of organisms). A recent metastudy (*13*) involving heterogeneous data from a large number of soil samples (>3000) inferred higher fungal diversity in hyper-arid and Arctic habitats compared to much of the tropics, challenging the common understanding about the global distribution of fungal diversity and of biodiversity in general (*5*, *7*, *14*–*16*).

While the biogeographic patterns seen in plants and animals are explained by competing hypotheses involving interactions between various factors of diversity (*16*), the joint effect of key drivers—water and thermal energy supply—on the global distribution of fungal alpha diversity has not been assessed. There is also a notable lack of global data providing joint analysis of other diversity aspects, including beta diversity (spatial variability of community composition), gamma diversity (regional species richness), and phylogenetic diversity (incorporating the evolutionary component of community diversity). Considering these diversity aspects together is pivotal for comprehending the spatial organization of biodiversity and becomes an imperative in development of sustainable ecosystem management strategies and effective biodiversity conservation schemes (*15*, *17*).

Here, we analyze global patterns of fungal taxonomic and phylogenetic alpha, beta, and gamma diversities based on long-read DNA sequencing of ca. 4000 topsoil samples (fig. S1), collected during six global surveys following comparable sampling and molecular analysis protocols: the Global Soil Mycobiome consortium (GSMc) (*6*), BIODESERT (*18*), MUSGONET and CLIMIFUN (*19*), GlobalAM (*20*), and GlobalWetlands (*21*). The samples encompass a wide variety of land cover types, including different types of woody and herbaceous plant communities, deserts, and agricultural and urban environments. To increase the discriminatory power of the analysis compared to previous works, we use the full-length internal transcribed spacer (ITS) region of the ribosomal RNA (rRNA) gene as a taxonomic marker (*22*, *23*). The resulting >14 million high-quality sequences are clustered into 800,000 operational taxonomic units (OTUs; fig. S2) using a 98% similarity threshold, a practical compromise for grouping sequences roughly corresponding to fungal species (*6*). Although the resulting OTUs may not always precisely represent distinct species, they serve as taxonomic proxies whose distribution patterns are likely similar to corresponding species.

While fungi exhibit immensely high functional diversity, they are, much like other organisms, commonly categorized by their most prominent roles in ecosystem functioning into pathogens, symbionts, and decomposers of dead organic matter. Leveraging the expert-curated FungalTraits database (*24*), we categorize species into functionally distinct, minimally overlapping groups (fig. S3 and table S1): arbuscular mycorrhizal (AM) and ectomycorrhizal (EcM) fungi, molds, nonmycorrhizal Agaricomycetes (NMA; mostly represented by saprotrophic macrofungi), and a group of species that are nonsymbiotically biotrophic on a wide variety of organisms (hereafter “pathogens”). We consider yeasts and nonyeast unicellular fungi that exhibit markedly different lifestyles from the predominant multicellular mycelial forms separately (*25*, *26*). Given their importance from a public health standpoint, we also distinguish opportunistic human pathogens (OHPs).

Drawing on these knowledge gaps and previous findings, we address the following questions. (i) What is the combined effect of precipitation and temperature on soil fungal alpha diversity? (ii) Do the global distribution and drivers of alpha diversity vary among fungal ecological groups, reflecting their niche attributes? (iii) What mechanisms underlie fungal species and phylogenetic diversity relationships? (iv) Is climate, through the effects on fungal species ranges (*9*, *13*), the primary driver of fungal compositional turnover (i.e., beta diversity)? (v) Is thermal energy supply, the main driver of animal and plant diversification (*27*), also the primary explanatory variable of fungal gamma diversity?

## RESULTS AND DISCUSSION

### Alpha diversity

**Fig. 1.**
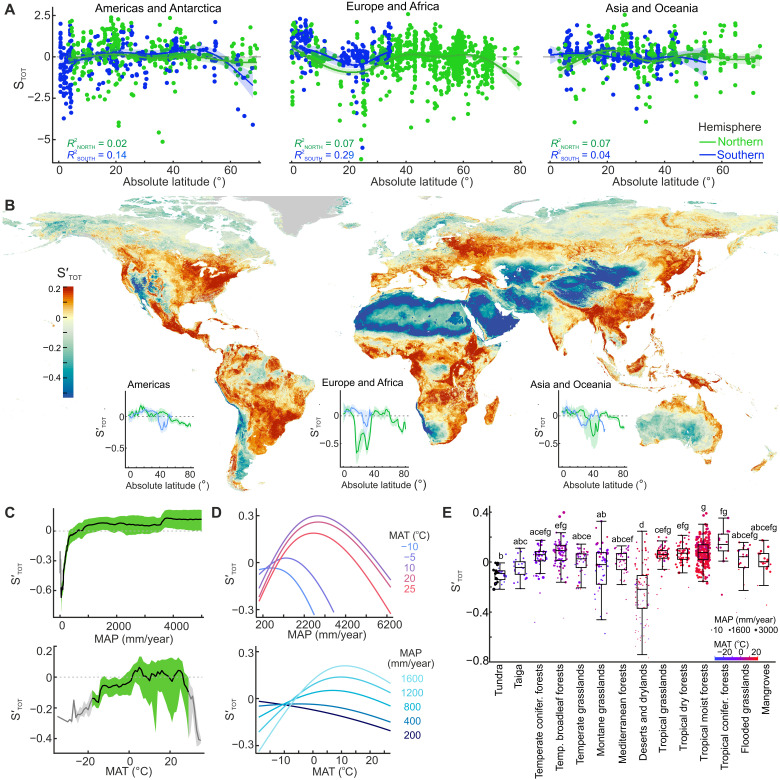
Global distribution of soil fungal alpha diversity. (**A**) Latitudinal distributions of total fungal alpha diversity (S_TOT_). (**B**) Consensus map of predicted total fungal alpha diversity (S′_TOT_), with insets showing S′_TOT_ latitudinal distributions in Northern (green) and Southern (blue) Hemispheres; areas covered with perennial ice are colored in gray. (**C**) S′_TOT_ (median and interquartile range) distribution in the global gradients of MAP and MAT; green and gray denote the predictions within and beyond training data coverage, respectively. (**D**) Conditional effects of MAP and MAT on S′_TOT_. (**E**) S′_TOT_ across terrestrial biomes, with dots showing median S′_TOT_ for 818 terrestrial ecoregions (*47*). Biomes sharing the same letter indicate no significant difference at the 5% level of significance after *P* value adjustment with the Holm-Bonferroni method.

The latitudinal distribution of the total number of fungal OTUs in the samples (i.e., alpha diversity, S_TOT_) exhibits peaks in tropical and temperate ecosystems, while showing depressions in desert, Arctic, and Antarctic ecosystems (Fig. 1A and fig. S4). To identify the factors explaining S_TOT_ variability, we used 135 publicly available climatic, vegetation, and edaphic variables (table S2). We eliminated correlated variables, prioritizing variables already identified as fungal biodiversity drivers in global and continental-scale studies, and then preselected 35 influential variables for modeling S_TOT_ distribution within each dataset using the extreme gradient boosting (XGBoost) machine learning technique. Using a consensus approach that accounts for model performance and representation of environmental conditions in the training data, we constructed a high-resolution global map of predicted S_TOT_ (S′_TOT_; Fig. 1B and figs. S5 to S9) with a 30–arc sec resolution equating to grid cells of <1 km^2^. The map highlights the largest fungal diversity “hotspots” (areas with S′_TOT_ above the global 97.5th percentile) in the East African highlands, Gulf of Guinea tropical forests, Appalachian forests, Central American dry forests, and Himalayan and New Guinean forests (fig. S10). The hotspots partly align with plant alpha diversity peaks (*14*), primarily along the equator, excluding the wettest parts of equatorial rainforests with S_TOT_ depressions. The S′_TOT_ “coldspots” (areas with S′_TOT_ below the 2.5th global percentile) include the Atacama, Sahara, and Arabian deserts, as well as the Central Asian drylands, reflecting the primary limiting effect of water deficit on soil fungal alpha diversity, similar to its impact on soil testate amoebae, earthworms, and other soil-dwelling organisms (*28*–*30*).

While S′_TOT_ displays a near-monotonic increase along the gradients of mean annual precipitation (MAP) and temperature (MAT) (Fig. 1C), corroborating previous works [(*5*, *7*, *12*, *30*), but see (*13*)], conditional effect analysis shows that S′_TOT_ response to both predictors varies substantially across climate types. Specifically, it is positively related to annual temperature in cold humid or temperate humid regions, and negatively so in hot or arid regions (Fig. 1D). The S′_TOT_ response to annual precipitation is positive in hot climates with up to ca. 2000 mm of annual rainfall, but negative in hyper-humid or cold areas. Correspondingly, while tropical and subtropical rainforests generally have high S′_TOT_ (Fig. 1E, fig. S11, and table S3), relatively lower values are predicted for the central wettest parts of the Amazon and Congo basins and for the Indo-Malay swamp forests, where hydric soils predominate. Across temperate broadleaf and mixed forests, S′_TOT_ peaks in the Balkans, Far East, and Appalachians, but has the lowest values in Western and Central European humid lowlands. In tundra and boreal forest biomes, S′_TOT_ peaks in the Far East and declines in subarctic and Arctic ecosystems.

According to the model predictions, land cover type also constitutes a major influencer of fungal alpha diversity, with deciduous forests supporting the highest S′_TOT_ in all biomes (table S3). At a broader scale, this effect is exemplified by S′_TOT_ gradients from steppes to transitional forest-grassland vegetation to forested biomes. The difference in S′_TOT_ between deciduous forests and either croplands or urban areas is particularly pronounced in the tropical dry forest biome. Consequently, agricultural expansion and urbanization, which cover over 30% of the biome area, lead to notable and extensive declines in S′_TOT_, as exemplified in the Deccan plateau, Central Indochina, and Central America. This reflects concerns about the loss of tropical biodiversity in other biotic groups (*15*). In addition, while less pronounced, yet extensive S′_TOT_ declines due to agricultural land transformation are also predicted in the Americas and Europe across Mediterranean and temperate forest biomes, as well as temperate grasslands.

The present analysis of the drivers of fungal diversity distribution is based on the most extensive global sample collected using a unified protocol and covering a large volume in the multidimensional space of environmental properties. However, certain habitats, characterized by unique combinations of environmental characteristics, have lower prediction precision and deserve future research focus (figs. S8 and S9). The large number of species forming the basis of our analyses supports the expectation of global fungal diversity (*31*), while its exceeding the number of currently named species underscores the necessity for additional efforts in describing mycobiota from various habitats (*32*).

### Alpha diversity of fungal ecological groups

**Fig. 2.**
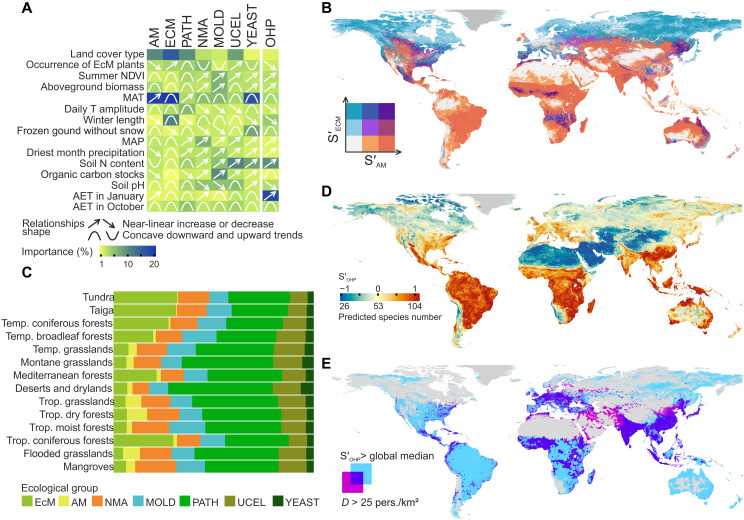
Alpha diversity of soil fungal ecological groups. (**A**) Importance of key predictors and the shape of their relationships with the local richness of fungal ecological groups. (**B**) Model-predicted global distribution of EcM and AM fungal local richness. Color legend denotes low, medium, and high EcM or AM fungal predicted richness; areas covered with perennial ice are colored in gray. (**C**) Functional profiles of soil mycobiota in different biomes based on predicted alpha diversity of fungal ecological groups. (**D**) Global distribution of predicted alpha diversity of OHP fungi and (**E**) overlap between areas with high predicted richness of OHP fungi and high human population density (*D*).

We examine and align the worldwide distributions of key fungal groups driving important soil processes on Earth through pathogenesis, symbiosis, and decomposition. We find that edaphic parameters, ecosystem productivity, and water availability are generally more important for saprotrophic groups, while temperature and vegetation composition are fundamental for mycorrhizal fungi (Fig. 2A and table S4). Conceptually mirroring previous findings (*13*, *33*, *34*), the latitudinal distributions of the predicted richness of AM and EcM fungi (S′_AM_ and S′_ECM_, respectively) are almost opposite (*r* = −0.54; fig. S12, A and B, and Supplementary Text) primarily divided by temperature niche (i.e., with optimal MAT >20°C for S′_AM_ and −15°C to +5°C for S′_ECM_; see fig. S13) and abundance of potential host plants. By overlaying the maps of S′_AM_ and S′_ECM_ (Fig. 2B), we outline two distinct areas with high S′_AM_ (generally in tropical forests and subtropical grasslands) and S′_ECM_ (in temperate and boreal forests) with transitional vegetation such as forest-grasslands, forest-steppes, and savannas, inhabited by species-rich communities of both groups.

While the diversity hotspots of molds and predominantly saprotrophic NMA are inferred, respectively, for temperate (e.g., in the Japanese Archipelago, Tasmania, and the Appalachians) and tropical (e.g., the Gulf of Guinea, Oceanian, and Central America forests) rainforests, S′_MOLD_ and S′_NMA_ are strongly correlated (*r* = 0.70) and respond very similarly to primary drivers (figs. S12 and S13). Molds, however, exhibit a stronger demand for soil carbon and nitrogen content and preference for more acid soils than NMA. Unlike other saprotrophic groups, yeast diversity is less responsive to MAP and nutrient availability. We predict relatively high S′_YEAST_ for arid alkaline soils, in agreement with the extremophilic habit of many yeast species and supporting the idea of an understudied yeast biota from extreme habitats (*25*, *35*). Conversely, nonyeast unicellular fungi exhibited narrower optimum ranges of climatic and edaphic parameters, and had predicted hotspots in temperate mesic forests but coldspots in arid regions and tropical hydric soils.

Compared to the other fungal groups, the effect of environmental variables (including land cover types) on S_PATH_ is weaker, resulting in a lower S′_PATH_ variability across the globe (fig. S12 and table S5). Primarily explained by diurnal temperature amplitude (DTA), S′_PATH_ is higher than average at DTA of ca. 7° to 13°C (i.e., optimal for most plants and ectotherms and fungal pathogens’ primary hosts) while sharply declining outside this range (i.e., in extreme hyper-arid, alpine, and Arctic habitats). These findings, summarizing the variety of relationships between pathogen diversity and host community properties (*36*–*39*), suggest that a biotic community exceeding a certain low threshold of diversity or biomass invariably shelters a relatively diverse community of fungal pathogens.

Our alpha diversity maps provide the basis for conserving fungal ecological groups and, thus, the ecosystem functions performed by them and the organisms reliant on them. Furthermore, the predicted functional profiles of soil mycobiota, built by summarizing the groups’ diversity, substantially vary across the globe (Fig. 2C). The unique functional profiles of terrestrial biomes and ecoregions (figs. S14 and S15) provide distinct ecosystem services and functions in global nutrient cycling, vividly exemplified by the contribution of mycorrhizal fungi in global carbon allocation (*4*).

Areas with high S′_OHP_ (Fig. 2D) cover the regions with the greatest human population density (Fig 2E). These results, while supporting the link between the frequency of OHP infections and social factors reported in epidemiological studies (*40*), may also point to an underexplored pathogenicity potential of fungal biota in less populated areas. In light of the elevated OHP potential in molds, we expect a larger number of yet undiscovered OHP species within S′_MOLD_ hotspots, where respective epidemiological surveillance is needed to mitigate public health risks.

### Phylogenetic alpha diversity

**Fig. 3.**
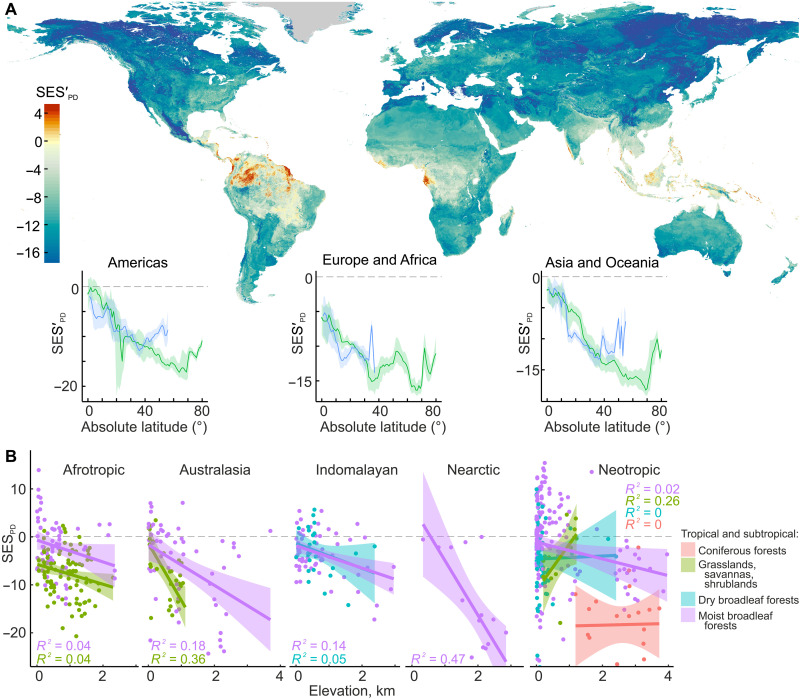
Global patterns in soil fungal phylogenetic structure. (**A**) Global distribution of predicted phylogenetic dispersion (SES′_PD_) of soil fungi, with accompanying latitudinal distributions of SES′_PD_ in the Northern (green) and Southern (blue) Hemispheres. (**B**) Association between SES_PD_ and elevation in tropical biomes. For clarity, the *y* axis is clipped, and two data points displaying low SES_PD_ values from the Neotropical biome are excluded.

Incorporating species relatedness into biodiversity estimates through phylogenetic diversity indices identifies the mechanisms of community assembly (*41*), and their potential functional value, and correspondingly underlies conservation prioritization (*15*, *42*). In fungal biogeography, phylogenetic diversity is rarely considered alongside taxonomic diversity [but see (*33*)]. In our dataset, the simplest measure of phylogenetic alpha diversity—total phylogenetic branch lengths in a community (S_PD_)—is tightly positively linked with species richness (*r* = 0.71 between S_PD_ and S_TOT-GSMc_). Accordingly, S′_PD_ broadly correlates with S′_TOT_ (fig. S17). Meanwhile, phylogenetic dispersion (SES_PD_), representing standardized difference between actual S_PD_ and S_PD_ of a randomly assembled community of the same richness, illustrates that approximately 93% of the sampled communities are phylogenetically clustered (SES_PD_ < 0). This highlights that environmental filtering for closely related, and thus functionally similar, species is the main global mechanism shaping local mycobiota. Phylogenetically overdispersed communities (SES_PD_ > 0), assembled with a greater contribution of competitive displacement among functionally similar species, are found in the equatorial tropics. Decreasing poleward (Fig. 3A) and with altitude in the tropical highlands (Fig. 3B), SES_PD_ is best explained by MAT (table S4). While SES_PD_ is independent of S_TOT_ (*r* = 0.01), it is affected by the richness of certain fungal groups in a community (positively by S_AM_ and S_UCELL_ and negatively by S_ECM_ and S_MOLD_; table S6).

The map of model-predicted SES_PD_ (SES′_PD_) shows strong latitudinal and elevational gradients of phylogenetic dispersion, mirroring plant and animal biogeographical patterns (*15*, *43*). On the basis of the known patterns in EcM and AM fungal evolutionary biogeography (*33*, *34*, *44*, *45*), the links between SES_PD_ and the richness of ecological groups highlight the impact of rapid speciation in specific genera of EcM fungi and the selective exclusion of AM fungi in colder climates on the global distribution of the phylogenetic structure of the whole soil fungal community. Research of the mechanisms involving unicellular fungi and molds, taxa with a largely unexplored evolutionary biogeography, represents a promising avenue for future investigations.

### Beta diversity

**Fig. 4.**
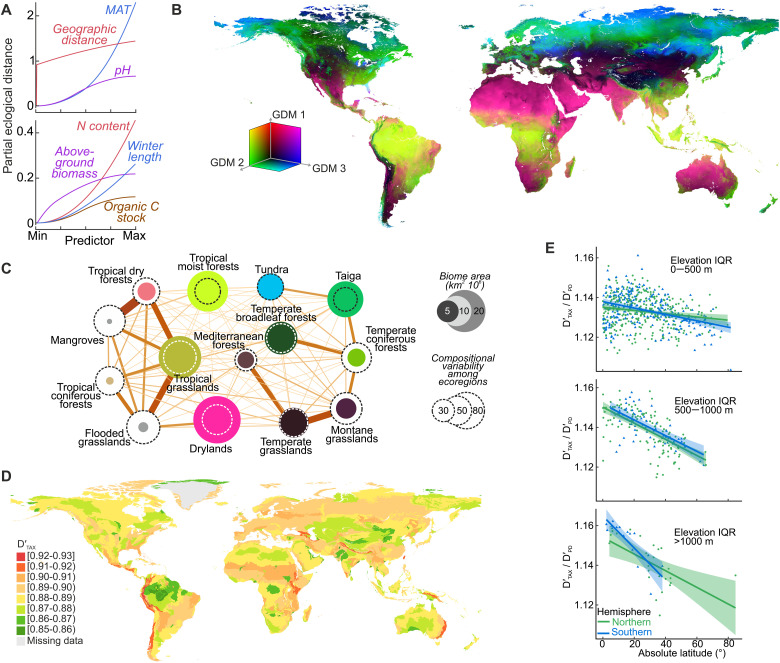
Global patterns of soil fungal beta diversity. (**A**) Compositional turnover of fungal communities over the ranges of the most influential variables: geographical distance (0.7 to 19,948 km), MAT (−11.8° to 29.3°C), soil pH (4.0 to 8.7), soil N content (0.4 to 16.7 g kg^−1^), aboveground biomass (0 to 1828 Mg C ha^−1^), organic C stocks (9.4 to 142.5 Mg ha^−1^), and winter length (0 to 365 days). (**B**) Predicted compositional dissimilarity of fungal communities, where color gradients represent species composition gradients derived from environmental predictors and combined into three GDM components; thereby, color similarity among locations in the map is proportional to compositional similarity of fungal communities. (**C**) Graph of compositional pairwise relatedness of terrestrial biomes, with node color indicative of the biome’s position in GDM components and edge width proportional to mycobiota compositional similarity between the biomes. (**D**) Predicted compositional dissimilarity among soil fungal communities within ecoregions (median of D′_TAX_). (**E**) Latitudinal distribution of ecoregional D′_TAX_-to-D′_PD_ ratio in areas with a small, medium, and large altitude difference (elevation interquartile range).

Beta diversity, measuring the compositional heterogeneity of biota among locations, is a crucial parameter for the development of strategies for regional- and continental-scale biodiversity maintenance (*17*). We examined global patterns of soil fungal beta diversity among GSMc samples based on Simpson’s pairwise dissimilarity in the composition of OTUs [D_TA__X_ (taxonomic dissimilarity)] and phylogenetic lineages [D_PD_ (phylogenetic dissimilarity)]. As the indices are strongly correlated (Mantel’s *r* = 0.86), their revealed drivers and distribution maps are largely similar (fig. S19). Specifically, while our sampling captures high compositional dissimilarity at the landscape scale, it also reveals the greater importance of the environment rather than geographical distance for fungal compositional variability at macroscales, supporting the results of the species distribution analyses (Fig. 4A) (*5*, *9*, *13*, *46*). The strongest environmental predictors of fungal spatial compositional turnover include MAT, soil pH, and N content (Fig. 4A and fig. S19).

We constructed maps of the model-predicted D_TA__X_ and D_PD_ (D′_TAX_ and D′_PD_, respectively) (Fig. 4B and fig. S20), which broadly illustrate a statistically significant divergence of all terrestrial biomes in mycobiota composition (Fig. 4C, fig. S18, and table S7), with tropical moist forests harboring the most unique communities. Mangroves, tropical coniferous forests, and flooded and mountain grasslands [i.e., small biomes associated with specific terrains “embedded” in large continuous biomes (*47*)] share the largest part of their fungal biota with surrounding biomes. However, given the specificity of such ecosystems, we consider that the uniqueness of their mycobiota could be underestimated in our study (and other ongoing works) due to the lack of variables accurately describing such habitats (as emphasized in mountain biodiversity studies) (*48*).

By averaging D′_TAX_ within terrestrial ecoregions (Fig. 4D) and a 150-km radius area, we predict that the most pronounced spatial turnover of soil fungi within subtropical and tropical regions arises from greater differences in altitude and, consequently, environmental heterogeneity (figs. S21 and S22). When accounting for the topography effect, we found that the D′_TAX_-to-D′_PD_ ratio declines with distance from the equator (β < −0.01, *P* < 0.001; Fig. 4E). In terms of tropical conservatism theory, this implies a nonlinear increase of tropical lineage loss with distance from the environmental optimum.

### Gamma diversity

**Fig. 5.**
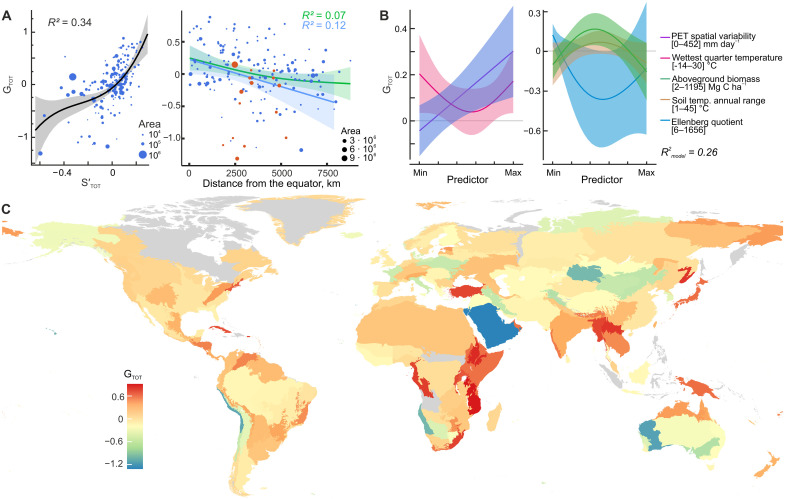
Gamma diversity of soil fungal communities. (**A**) Relationships of total fungal regional richness (G_TOT_) with median S_TOT_ and distance from the equator. Separate regression lines are shown for the Northern (green) and Southern (blue) Hemispheres. Deserts and drylands (red dots) are excluded from the regression analysis. (**B**) Marginal effects of the significant predictors on G_TOT_. Potential evapotranspiration (PET) spatial variability is the interquartile range of Thornthwaite’s PET, and the Ellenberg quotient, the ratio of mean temperature of the warmest month to annual precipitation, is an index of drought. (**C**) Map of G_TOT_ at the ecoregion scale (*46*).

Fungi are estimated to comprise 2 million to 3 million species (*31*), with the global distribution of regional species richness remaining one of the least studied aspects of fungal diversity. On the basis of our global surveys and museum collections’ sequences deposited in the UNITE (including GenBank) database (*49*), we evaluate gamma diversity as OTU richness in a superecoregion [as defined by (*46*)], accounting for variations in sampling and sequencing effort (table S8). The overall fungal gamma diversity (G_TOT_) is linked to regional average S′_TOT_ (Fig. 5A) and, correspondingly, responds to the index of drought (Fig. 5B). The strong positive effect of spatial variability of thermal energy supply (i.e., of potential evapotranspiration) on G_TOT_ matches the classical positive relationships between environmental heterogeneity and regional plant and animal biodiversity (*50*). Given that temperature is the main driver of compositional turnover and SES_PD_, the revealed effect of energy supply variability on G_TOT_ also links regional richness with beta diversity and phylogenetic dispersion. The significance of the annual range of soil temperature for G_TOT_ suggests that the relationships between physiological tolerance to temperature fluctuations and distribution ranges of plant and ectothermic species (*51*) also apply to fungi. Meanwhile, MacArthur and Wilson’s (*52*) classic theory of island biogeography does not hold true for our dataset, as G_TOT_ is not related to island area or distance from mainland (table S9).

In the G_TOT_ map, much like the situation in other kingdoms (*48*, *53*, *54*), most of the G_TOT_ peaks in the tropics are located not only in mountainous regions such as the eastern African highlands, New Guinea, and eastern Himalayas but also in coastal lowlands in Central Africa and Australia (Fig. 5C). Notably, the eastern slopes of the tropical Andes, a region known for its high plant and vertebrate richness (*48*, *53*, *54*), are not among the identified areas with high fungal G_TOT_. In the temperate zones, G_TOT_ peaks in northeastern United States, Anatolian forests, the Japanese Archipelago, and Ussuri forests. Beringia exhibits the highest gamma diversity among Arctic superecoregions, possibly because of its relatively long glacier-free history (*55*). The lowest G_TOT_ values are observed in desert areas.

While regional richness of fungal ecological groups generally coincides with alpha diversity, peaking in the same geographical regions and largely driven by similar variables (figs. S24 and S25, tables S10 and S11, and Supplementary Text), gamma diversity of pathogens is also regulated by the regional variability of climatic predictors and elevation, which highlights the importance of environmental heterogeneity for this wide-ranging ecological group.

### Synthesis

Together, the results of our multidimensional analyses indicate that, alongside other kingdoms, soil fungi exhibit peaks in alpha, beta, and gamma diversities in tropical (mostly mountainous) regions while decreasing toward the poles and abruptly dropping in hyper-arid regions. While water deficit in drylands is an evident limiting factor for alpha diversity, our models incorporating water-energy balance also explain the declines in local diversity in tropical hyper-humid soils—one of several unresolved issues in fungal biogeography. In addition, phylogenetic and species turnover are linked to gamma diversity by spatial variability in thermal energy supply. By integrating the distribution patterns of taxonomic, phylogenetic, and ecological group diversities, we outline the mechanisms shaping soil fungal communities across environmental gradients and space and highlight the pivotal roles of EcM and AM fungi, molds, and nonyeast unicellular fungi in the mechanisms. One of our critical findings is that the predicted functional uniqueness of soil mycobiota across the majority of terrestrial ecoregions implies their role as functional units with different roles in global nutrient cycles. The potential cross-regional impacts of the loss of such functional units emphasize the imperative of collaborative conservation efforts that aim to preserve all types of biota to safeguard biosphere functional integrity. Considering the heightened vulnerability of endemic species to environmental changes, we propose assessing the conservation value of local mycobiota based not only on its diversity but also on its compositional uniqueness. The models and high-resolution maps provided by our work represent the basis for conservation planning (of fungi or groups dependent on them) and for considering soil fungi in explanatory and predictive modeling of macroecological, biogeochemical, and epidemiological processes.

## MATERIALS AND METHODS

### Datasets

#### Fungal surveys

The GSMc (https://GSMc-fungi.github.io/) dataset (*6*) was the basis for the analysis of taxonomic and phylogenetic alpha, beta, and gamma diversities conducted. This dataset comprises fungal sequence data from 128,000 subsamples at 3200 study sites collected from all continents in 2010–2019 following strict sampling and analytical protocols. In short, 40 soil cores (5 cm in diameter and 5 cm in depth) were collected from a 2500 m^2^ area at each study site and were pooled. For gamma diversity and validation of alpha diversity analyses, we used the data from five additional global surveys applying comparable sampling protocols: BIODESERT (249 samples) exploring dryland ecosystem response to grazing pressure and climate change (*18*); MUSGONET and CLIMIFUN (290 samples) (*19*); GlobalAM (199 samples), assessing arbuscular mycorrhizal fungal diversity worldwide (*20*); and GlobalWetlands (85 samples) (*21*). A map displaying the sampling locations is shown in fig. S1. Because all sampling plots in all the surveys are of a comparable size, the design excludes the “spatial grain effect” [i.e., nonlinear relationships between species richness and sampling plot area (*14*, *50*)]. The effect of environmental properties (e.g., microtopography and local plant richness) and habitat type on the microscale spatial variability of soil fungal community composition has been documented. While we fully acknowledge that taking microscale variability into account in diversity assessment can enhance the accuracy of the analyses, the current affordability of high-throughput molecular-genetic tools does not permit the processing of such a large number of samples. Thus, the present study operates on the assumption that the sampling effort (number of bulked subsamples) adequately captures local fungal richness across the studied habitats.

When necessitated by local authorities, the appropriate permissions were secured for the execution of field work. No species that are classified as endangered or protected were involved in the sampling process or adversely affected. For experimental blinding, each soil sample was assigned a unique identifier used in all subsequent analyses, ensuring that the research team remained unaware of the geographic origin of the samples, thereby reducing potential location-based bias.

#### Environmental data

To model the diversity of soil fungal communities across diverse ecosystems, we used a comprehensive collection of environmental datasets (table S2). These datasets encompassed a wide range of climatic, edaphic, and biotic factors, including the CHELSA (*56*), ENVIREM (*57*), CMCC-BioClimInd (*58*), SBIO (*59*), TerraClimate (*60*), SoilGrids (*61*), and other relevant datasets. By incorporating variables such as temperature, precipitation, evapotranspiration, soil properties, vegetation indices, land cover, and plant species richness, our analyses aimed to capture the complex interplay of factors that drive fungal diversity patterns.

To infer regional diversity patterns, we used 818 ecoregions cf. Olson *et al.* (*62*) based on the revised map of Dinerstein *et al.* (*47*) and 174 superecoregions defined by Tedersoo *et al.* (*46*).

### Molecular and bioinformatic analyses

DNA extraction was performed using different methods for each of the above-described surveys (see the methods of each survey for details). Polymerase chain reaction and amplification were performed identically for DNA samples obtained from all studies following the protocol of Tedersoo *et al.* (*6*). To obtain full-length sequences of the ITS region, we used the universal eukaryotic primers ITS9mun (3′-GTACACACCGCCCGTCG-5′) and ITS4ngsUni (3′-CCTSCSCTTANTDATATGC-5′) (*63*). Both primers contained unique 12–base pair indices used for sample multiplexing. Library preparation and DNA sequencing were performed using the PacBio Sequel II System (Pacific Biosciences, Palo Alto, USA) as described previously (*6*). The DNA sequences obtained were demultiplexed to samples with LIMA v2.0.0 software (Pacific Biosciences) and quality-filtered with VSEARCH v2.17.0 (*64*). All reads with more than one ambiguous nucleotide or more than two expected errors were eliminated during quality filtering. To keep only the ITS region, SSU and LSU regions were trimmed using ITSxpress v1.8.0 (*65*). Sequences were clustered into OTUs (*66*) at 98% sequence similarity and taxonomically annotated using the BLASTn method with BLAST+ v2.11.0 against the UNITE database v9 (*49*) (https://unite.ut.ee/) to represent roughly species-level entities. More details on bioinformatic analyses are provided by Tedersoo *et al.* (*6*). The bioinformatic analysis involved the use of multiple scripts, which were primarily incorporated into the open-source pipeline NextITS (*67*). This pipeline is available for public use and can be accessed on the GitHub platform (https://Next-ITS.github.io/).

We acknowledge that sequence clustering at the chosen similarity threshold reflects an overall compromise across different taxonomic groups. It results in the inflation of species richness of some taxa and the underestimation of some species-rich groups of Ascomycota with slowly evolving ITS regions (e.g., in certain families in Helotiales, Hypocreales, and Eurotiales). However, due to practical limitations, applying different clustering thresholds to distinct fungal groups is currently unfeasible (*68*).

We used the FungalTraits database v1.3 (*24*) to categorize fungal species into functional groups based on their ecological and morphological characteristics. These ecological groups are as follows: (i) AM fungi (3.7% of total OTUs), including all Glomeromycota but excluding all Endogonomycetes due to the lack of data separating AM species from free-living species; (ii) EcM fungi (15.4%); (iii) non-EcM Agaricomycetes (9.3%), primarily saprotrophic macrofungi; (iv) molds (5.8%), including Mortierellales, Mucorales, Umbelopsidales, Aspergillaceae and Trichocomaceae of Eurotiales, and *Trichoderma* of Hypocreales; (v) putative pathogens (11.4%), consisting of plant, animal, and fungal pathogens as primary or secondary lifestyles; (vi) soil borne OHPs (7.5%), excluding Mortierellales; (vii) yeasts (1.3%), excluding dimorphic yeasts; and (viii) other unicellular, nonyeast fungi (9.6%), including Chytridiomycota, Aphelida, Rozellomycota, and other early-diverging fungal lineages. Refer to table S12 for data structure details.

### Statistical analyses

All statistical analyses were carried out in R v4.3.0 unless otherwise stated. Data handling was performed using the data.table v1.14.2 and Apache Arrow v10.0.0 packages. Data visualization was done using the ggplot2 v3.3.6 package.

#### Alpha diversity

In our study, we evaluated fungal OTU richness in samples (denoted as S). This estimation was conducted across all fungal OTUs (S_TOT_) as well as for distinct ecological groups of fungi (indicated with subscripted labels corresponding to an ecological group name; e.g., S_EcM_ and S_AM_ for ectomycorrhizal and arbuscular mycorrhizal fungi). As an alpha diversity metric robust to sequencing depth variability between samples, we used the residuals from a linear regression of the logarithmically transformed number of fungal OTUs against the logarithm of the sequencing depth (figs. S26 and S27 and table S13). We chose not to use the widely applied alternative method of rarefying abundance tables, as it discards substantial amounts of data.

For fungal phylogenetic alpha diversity assessment, we used the sum of the total length of phylogenetic branches in a community (S_PD_) and sequencing depth normalized with ranked subsampling (SRS) (*69*). To construct a phylogenetic tree, we followed the method outlined in (*70*). This method entails the use of a phylogeny built from nearly complete rRNA gene sequences (18*S* and 28*S*), calibrated to divergence time, which served as a backbone tree for OTU assignment using sequence classification information. As a measure of phylogenetic dispersion in fungal communities, we estimated standardized effect size of S_PD_ (SES_PD_) using PhyloMeasures v2.1 (*71*), which was calculated as the deviation of the observed community’s S_PD_ from the S_PD_ of a community under a null model of random species distribution.

We used XGBoost to model the relationship between OTU richness or phylogenetic diversity and explanatory variables, which included bioclimatic (*56*–*58*, *60*, *72*), edaphic (*59*, *61*, *73*, *74*), and vegetation-related predictors (*53*, *75*–*77*). Table S2 lists the variables included in the analysis. A mask created from Copernicus Global Land Cover layers v3 (*75*) was used to restrict the mapped areas to land not covered with glaciers and to exclude permanent inland water bodies. Spatial autocorrelation assessment for response variables and predictors was performed across different geographical distance thresholds using Moran’s *I* index. In general, geographical distance had negligible effects on alpha diversity (maximum Moran’s *I* = 0.25 at the 100-km distance class). To avoid an excessive number of variables and reduce multicollinearity in the predictors, we excluded highly correlated variables (Pearson’s *r* > 0.8 and variance inflation factor > 10) using spatialRF v1.1.4 (*78*) and retrieved the most relevant candidate variables for modeling using Boruta v7.0.0 (*79*). Models were fitted using xgboost v1.6.0.1 (*80*) via mlr3 v0.13.3 framework (*81*). To avoid overfitting, we used nested resampling (*82*) with fivefold cross-validation for hyperparameter tuning and evaluation of model performance. Predictor importance scores were estimated as the average gain in model accuracy brought by the predictor across all decision tree splits where this predictor was used. To incorporate the spatial autocorrelation signal, we calculated model residuals at the sampling sites and used inverse distance weighting to interpolate residuals beyond the sampling sites. To obtain final alpha diversity predictions, interpolated residuals were added to the model-based predictions (*83*). To delineate the contribution of each predictor in the prediction of fungal richness for individual grid cells, we used Shapley values, which were computed using the fastshap package v0.0.7 (*84*). We used terra v1.6-7 (*85*) to generate the fungal diversity maps. The maps were constructed at a spatial resolution of 30 arc sec, which roughly corresponds to raster grid cells each covering a median area of 0.67 km^2^ (interquartile range 0.47 to 0.80 km^2^). Although we collected samples in Antarctica, we excluded this region from predictive modeling due to the lack of available data for numerous predictors. The rasterVis package v0.51.5 (*86*) was used for visualization. The creation of correlation matrix graphs was carried out using corrplot v0.92 (*87*).

The GSMc-based alpha diversity analyses were validated by performing similar analyses using the GlobalAM, BIODESERT, and CLIMIFUN + MUSGONET datasets. To assess the area where model predictions are confined within the observed ranges of environmental variables, we estimated the dissimilarity index DI (*88*) for each dataset. DI measures a variable-importance-weighted distance of environmental conditions on the map to the values observed in the training data. By combining four maps, weighted according to their model performance and the inverse of DI for each location, we generated a consensus map of fungal alpha diversity. DI was also used to delineate the area of applicability (*88*) of the predictions (fig. S9). To estimate the prediction uncertainty of diversity estimates, we used a 10-fold cross-validation approach and used standard deviation (SD) as a measure of uncertainty (fig. S8). To identify regions of exceptionally high (hotspots) and low (coldspots) species richness, we determined the top and bottom 2.5% quantiles of the richest and poorest grid cells on the map (*89*).

To assess the interaction of MAP and MAT as well as their relative impact on the total richness of fungal communities, we fitted a generalized additive model (GAM) with tensor product smooth functions with three knots using mgcv package v1.8-41 (*90*).

#### Beta diversity

For beta diversity analyses, we excluded OTUs with <10 occurrences as well as samples with <10 OTUs. To estimate the taxonomic distinctness (D_TA__X_) of fungal communities, we used Simpson’s pairwise dissimilarity index(the proportion of unique species found in a smaller community from a larger community richness), because, unlike other metrics, it is unaffected by differences in OTU richness between samples (*91*). Phylogenetic dissimilarity (D_PD_) was calculated using the same equation but based on shared and unique branches of the phylogenetic tree (*92*). Estimation of phylogenetic beta diversity was performed using phyloregion package v1.0.6 (*93*). To estimate the correlation between D_TA__X_ and D_PD_ matrices, we used Mantel’s permutation test based on Pearson’s correlation coefficient. For the ordination of samples, we performed principal coordinates analysis using the “capscale” function of the vegan v2.6-2 (*94*) package.

Predictors of fungal compositional turnover were assessed for all fungi using generalized dissimilarity modeling (GDM) and permutational analysis of variance [PERMANOVA; (*95*)] approaches as implemented in the gdm v1.5.0-3 (*96*) and vegan packages, respectively. Predictors’ relative importance in GDM models was quantified using a permutational test (1000 iterations) by estimation of the percent change in the explained deviance between a model fit with and without a permuted predictor. In addition, the relative importance of the variables included in the GDM was ranked according to the I-spline values rescaled to range between 0 and 1. Fungal compositional centroids of ecoregions were found using principal coordinates based on the GDM model. To evaluate local compositional turnover (fig. S21), we calculated and plotted the average expected compositional dissimilarity of each location and its closest neighbors within a 150-km radius. To assess the similarity among soil fungal communities at the ecoregional level, we conducted an ordination using the centroids derived from principal components analysis components based on the GDM-transformed environmental predictors.

The within-biome compositional variability of fungal communities was estimated by calculating the median distance of ecoregions to the biome centroids. To construct a graph representing the pairwise relatedness of fungal communities among biomes, we used Gephi v0.10 (https://gephi.org/). Compositional similarity between biomes was determined using the inverse of a pseudo-*F* statistic obtained from the PERMDISP analysis (*97*), which involved 10,000 randomization permutations.

#### Gamma diversity

For gamma diversity, the above datasets were augmented with the GlobalWetland survey (*21*) (85 samples) and Sanger-sequenced sites from the UNITE database (7706 samples). Logarithmically transformed cumulative ecoregion species richness was subjected to model selection against the logarithmically transformed number of samples and sequencing depth to calculate residual richness. To infer the relationship between fungal gamma diversity and environmental predictors, we used a GAM model with penalization and thin plate regression splines with a basis dimension of 3. For preselection of the most important predictors, we followed the same algorithm as for alpha diversity analysis. To extract the model coefficients, we used the parameters package v0.20.1 (*98*). Maps were constructed using the sf v1.0.8 package (*99*) and QGIS v3.28 (https://qgis.org/).
