# Supplementary material for: Connecting the multiple dimensions of global soil fungal diversity
Source: Sci Adv. 2023 Nov 29;9(48):eadj8016. doi: 10.1126/sciadv.adj8016 (PMC10686567; doi:10.1126/sciadv.adj8016)
Supplement: Supplementary file 1 — Supplementary Text Figs. S1 to S28 Legends for tables S1 to S13 [file sciadv.adj8016_sm.pdf]

Science Advances
AAAS

Supplementary Materials for

Connecting the multiple dimensions of global soil fungal diversity

Vladimir Mikryukov *et al.*

Corresponding author: Vladimir Mikryukov, vladimir.mikryukov@ut.ee; Leho Tedersoo, leho.tedersoo@ut.ee

Sci. Adv. **9**, eadj8016 (2023)
DOI: 10.1126/sciadv.adj8016

The PDF file includes:

Supplementary Text
Figs. S1 to S28
Legends for tables S1 to S13

Other Supplementary Material for this manuscript includes the following:

Tables S1 to S13

Supplementary Text

Alpha diversity of fungal ecological groups

Latitudinal distributions of the expected richness of EcM and AM fungi (S'_{EcM} and S'_{AM}, respectively) were nearly opposite and primarily explained by land cover and temperature (figs. S5 and S11), with an optimal mean annual temperature (MAT) >20 °C for S'_{AM} and -15 °C to +5 °C for S'_{EcM} (fig. S13). Consequently, in concordance with the general patterns of global diversity distributions of AM fungi, the largest hotspots of AM fungal diversity are predicted across the Indian subcontinent and in certain ecoregions of Sub-Saharan savanna, whereas tundra and the Siberian and Alaskan taiga support the lowest S'_{AM}. Conversely, S'_{EcM} peaks in the temperate and boreal forests, with large diversity hotspots in the Far East, Western Mediterranean region, and North American temperate coniferous forests, supporting the previously revealed inverse LDG for EcM fungi (*5, 35, 11*). The distributions of S'_{AM} and S'_{EcM} outline two distinct global areas with high alpha diversity for each (Fig. 2B, fig. S12). However, in smaller regions with transitional vegetation like wet prairies, forest-steppes, and savannas, there are rich local communities of both EcM and AM fungi.

Diurnal temperature amplitude (DTA, bio02) and land cover type were the best predictors of the alpha diversity of pathogenic fungi, with S'_{PATH} declining at DTA < 7 °C, i.e., in tropical lowland swamps, and at DTA > 13 °C – in hyper-arid regions, arctic and alpine tundra (fig. S12C). Despite the significant effect of land cover, a lower difference between woody and herbaceous vegetation in S'_{PATH} is expected compared to the other groups (table S3). The coldspots of pathogenic fungal diversity were predicted in the Indomalayan islands, while large hotspots were identified in Indomalayan continental rainforests and in the Ethiopian Highlands.

The strongest predictors of non-mycorrhizal Agaricomycetes alpha diversity (S'_{NMA}) were MAT, mean annual precipitation (MAP, bio12), and soil pH. S'_{NMA} peaked at -6°C and 25°C (fig. S13) and was positively related to MAP and soil acidity. Critical values of soil organic carbon stock and nitrogen content for the group diversity were estimated at 4 kg m^{-2} and 2.5 g kg^{-1}, respectively. Non-mycorrhizal Agaricomycetes diversity hotspots were predicted for Oceanian islands, Central America, and the Gulf of Guinea forests, but coldspots covered much of the hyper-arid regions (fig. 12D).

Edaphic properties were among the main predictors of mold alpha diversity. Consistent with their copiotrophic lifestyle, S'_{MOLD} almost linearly increased in the gradient of carbon supply (fig. S7) and had the greatest (compared with the other groups) demand for soil nitrogen content (with critical lower value of ca. 3 g kg^{-1}). Diversity hotspots of molds are located in humid coastal and island temperate and tropical forests at low soil pH (≤5.5 units) such as the Japan Archipelago, Great Britain, Eastern Australia, Tasmania, New Guinea, and the Appalachians (fig. 12E).

The largest hotspots of yeast diversity were recovered in warm temperate forests of Europe, eastern North America, Tasmania and eastern Australia, and Valdivian forests. The main coldspots were found in the arctic tundra, sand deserts, and certain forest ecoregions along the Northern Tropic.

The most favorable conditions supporting a high diversity of unicellular fungi included >600 mm MAP, positive MAT, and nitrogen-rich soils with 4.5 < pH < 8 (fig. S13). Correspondingly, the highest S'_{UCELL} values were predicted for temperate mesic forests (fig. 12G), while coldspots were identified in hyper-arid soils and tropical hydric soils.

The regions with high expected alpha diversity of opportunistic human pathogens (S'_{OHP}) are well delineated by actual evapotranspiration in January and soil nitrogen content of ca. 1-3 g kg^{-1} (fig. S28). Consequently, S'_{OHP} peaks in tropical areas receiving more than 1,000 mm MAP, with the global diversity hotspots in Central America, South Africa and Madagascar, New Guinea, Southwest Australia, and Northern Triangle with surrounding forested ecoregions (fig. 12H), while the coldspots are expected in Palearctic deserts and polar Nearctic.

Gamma diversity of ecological groups of fungi

AM fungal G (G_{AM}) peaks in tropical regions such as the Brazilian and African savannas and wooded drylands of Oman. G_{AM} increases with wettest-quarter temperature, soil bulk density, and spatial variability of actual evapotranspiration in May, but decreases with distance from the equator (R^2_{model} = 0.40).

EcM fungi (G_{EcM}) are regionally diverse in the Far East, Svalbard, northeast Australia, South-Central Africa, northwest Canada, and southernmost Patagonia. The lowest G_{EcM} occurred in desert areas (excepting the Sahara) and much of South America. G_{EcM} distribution was primarily described by positive relationships with distance from the equator, EcM plant occurrence, and within-ecoregion heterogeneity in January soil moisture content (R^2_{model} = 0.19).

Non-mycorrhizal Agaricomycetes had the highest G (G_{NMA}) in tropical and subtropical forests including much of Central Africa, Central America, Southeast Asia, New Guinea, Japan, and the Atlantic rainforest of Brazil. G_{NMA} increased towards higher temperature of the wettest quarter, and moderate levels of soil carbon stock and spatial variability of soil moisture in May (R^2_{model} = 0.42). G_{MOLD} (R^2_{model} = 0.33) and G_{OHP} (R^2_{model} = 0.31) peaked in the East Beringia, Amazonia, New Guinea, and the Appalachians, showing positive links with aboveground biomass and soil acidity. Gamma-diversity of yeasts (G_{YEAST}) was positively linked with MAT, MAP, and maximum monthly temperature, peaking in Central Africa and western South America (R^2_{model} = 0.17). Distribution of non-yeast unicellular fungi (G_{UCELL}) was primarily reflected by soil carbon stock, MAT, and actual evapotranspiration in May (R^2_{model} = 0.26), with top scores in East African highlands, Northwest Russia, Northeast USA, and Taiwan.

The highest regional richness of pathogenic fungi (G_{PATH}) was registered in the subtropics and tropics, with diversity hotspots in the Anatolian forests, and East and South Africa. G_{PATH} distribution was primarily explained by regional topography, December-to-March NDVI, plant richness, and variability of energy and water-energy climatic parameters (R^2_{model} = 0.29).

Supplementary Figures

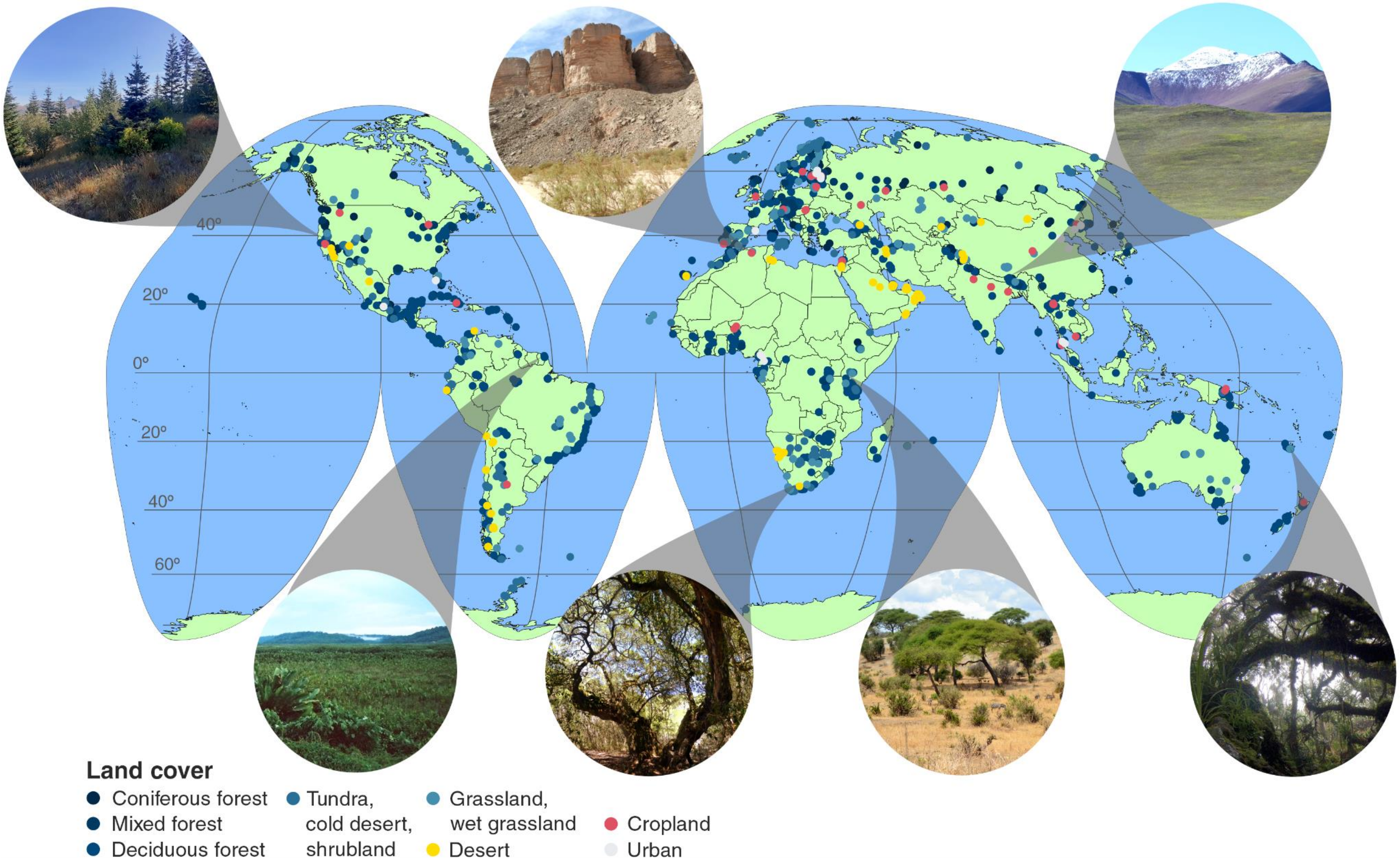

Fig. S1. Geographic distribution of study sites.

The map, presented in Goode's homolosine projection, shows the exact locations where the research was conducted, each marked by a dot. The different colors of the dots signify various types of land cover. Inset photos, captured by Sten Anslan and Sergei Põlme, provide visual representation of some of these sampling sites.

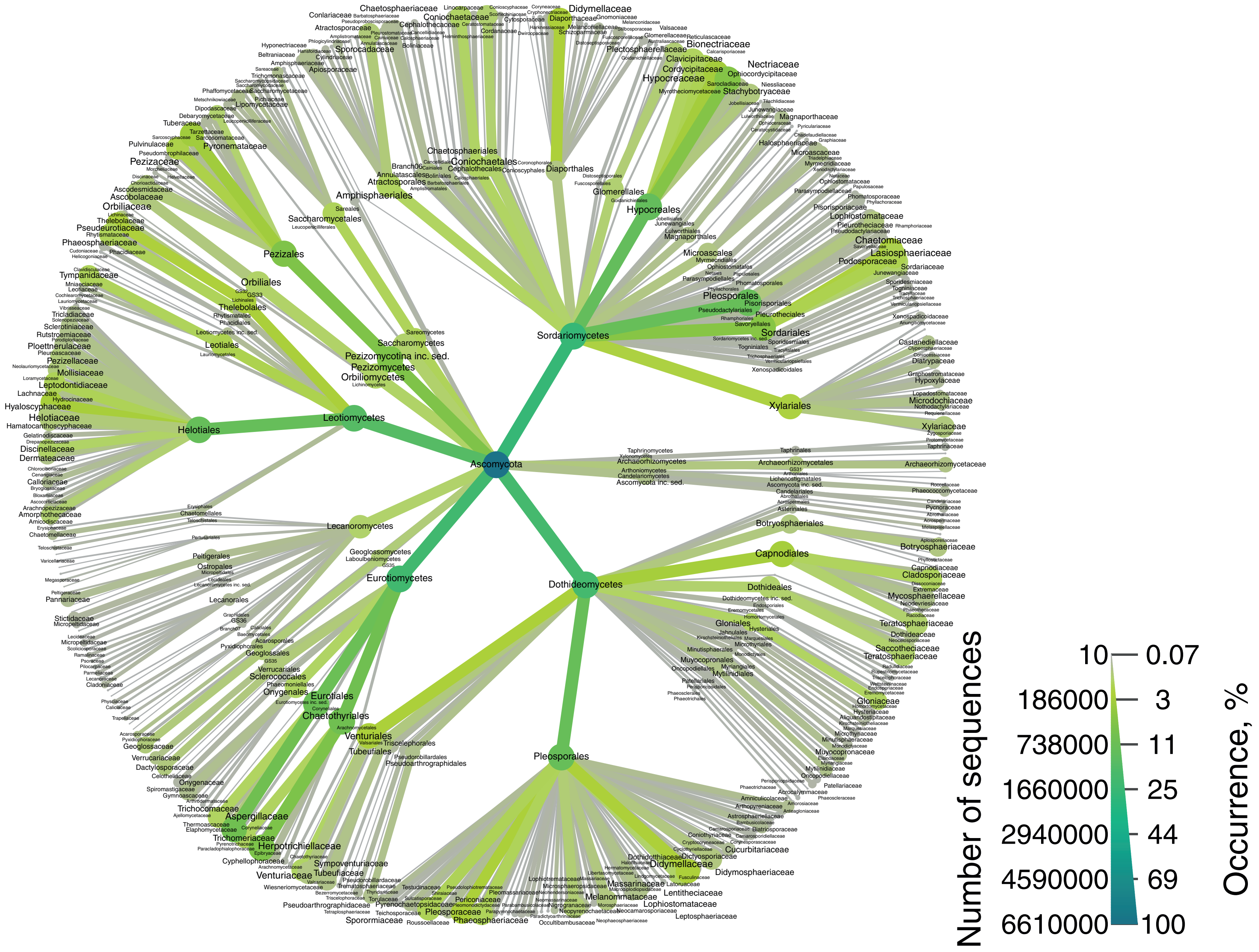

Fig. S2. Global taxonomic diversity of soil fungal communities as revealed by PacBio sequencing of the full-length *ITS* region.

(**A**) Phylum Ascomycota. Node size denotes the occurrence of taxa, the color is proportional to the number of reads.

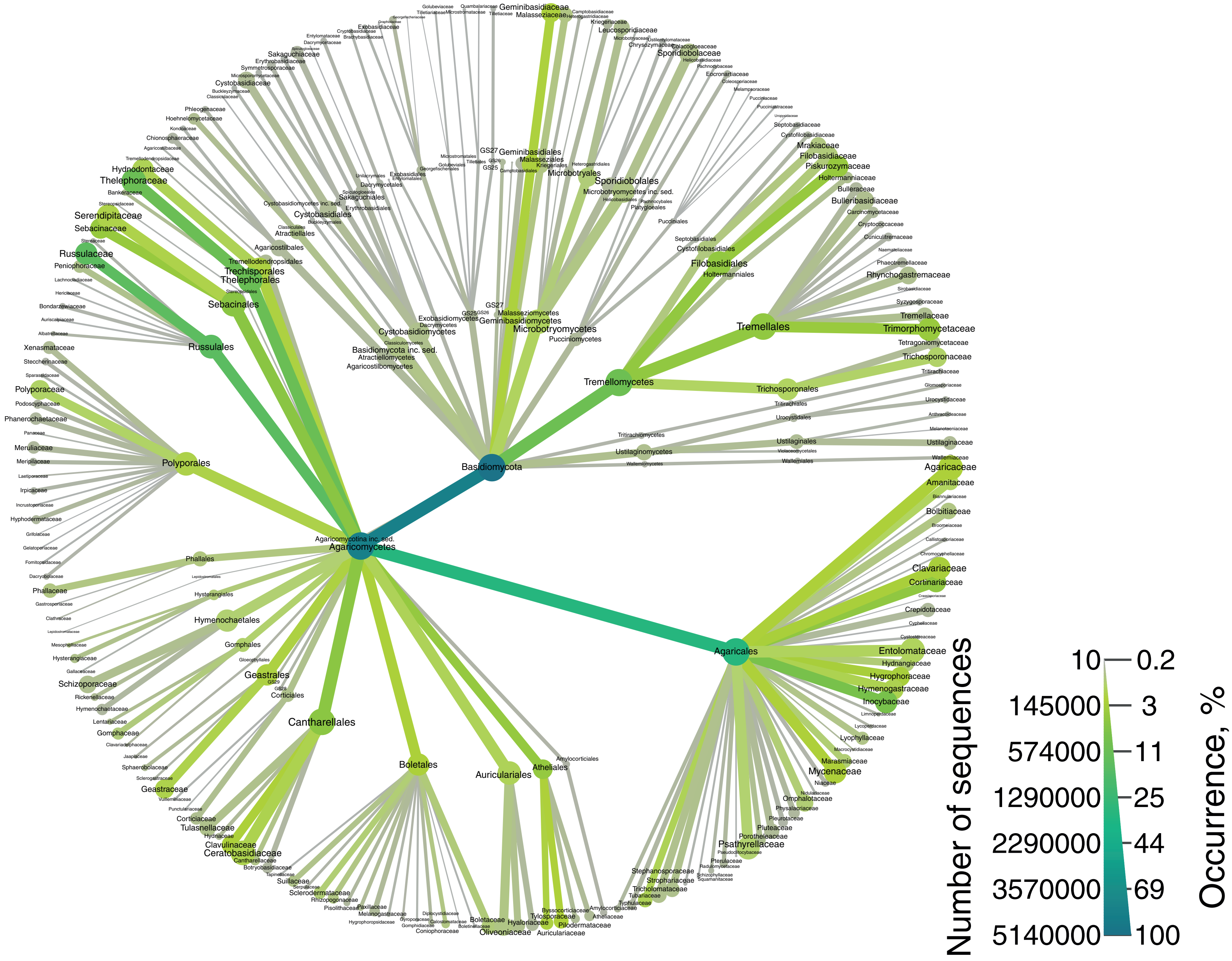

Fig. S2. Global taxonomic diversity of soil fungal communities as revealed by PacBio sequencing of the full-length *ITS* region.

(**B**) Phylum Basidiomycota. Node size denotes the occurrence of taxa, the color is proportional to the number of reads.

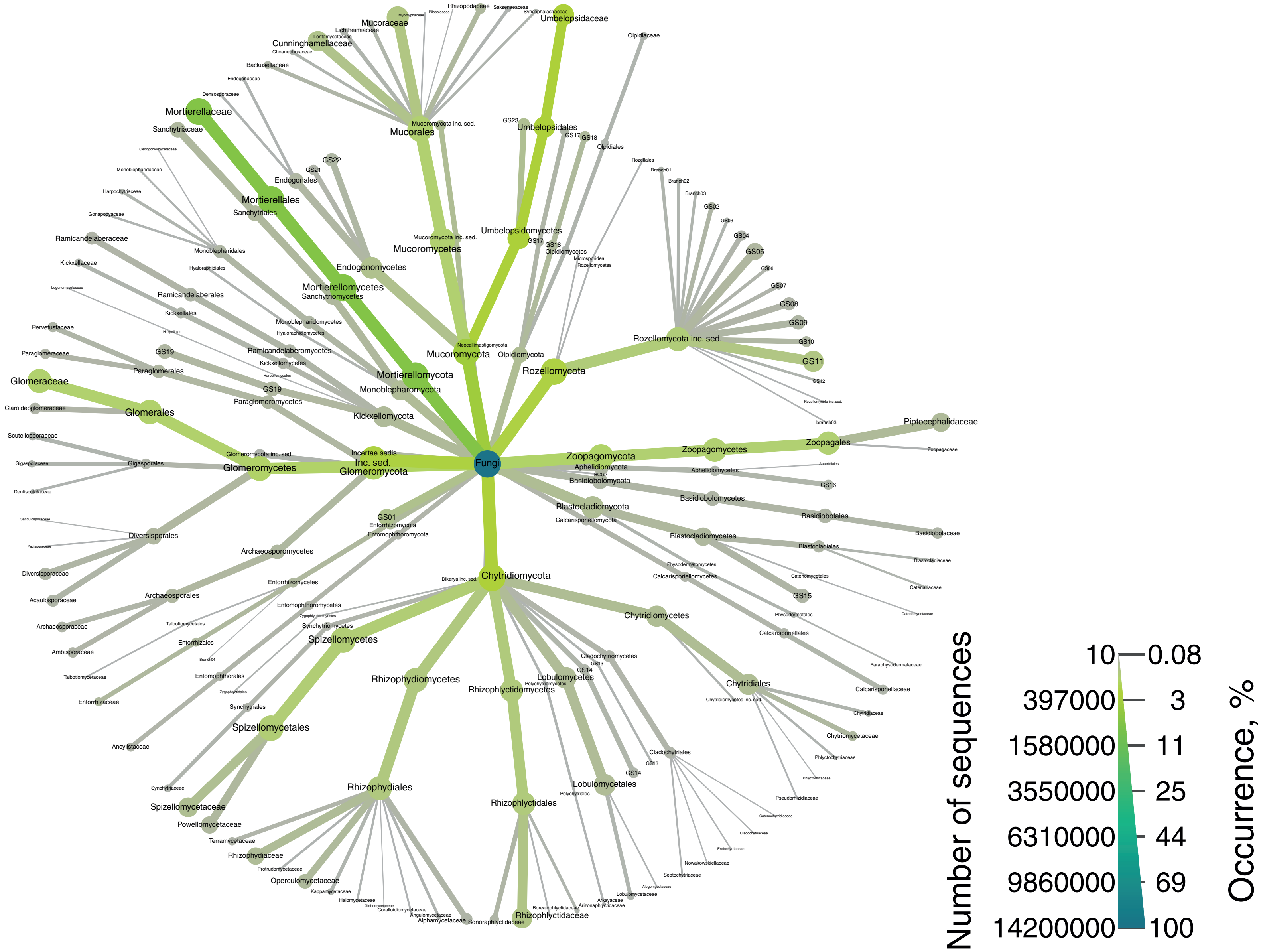

Fig. S2. Global taxonomic diversity of soil fungal communities as revealed by PacBio sequencing of the full-length *ITS* region.

(**C**) The other fungal phyla. Node size denotes the occurrence of taxa, the color is proportional to the number of reads.

A

B

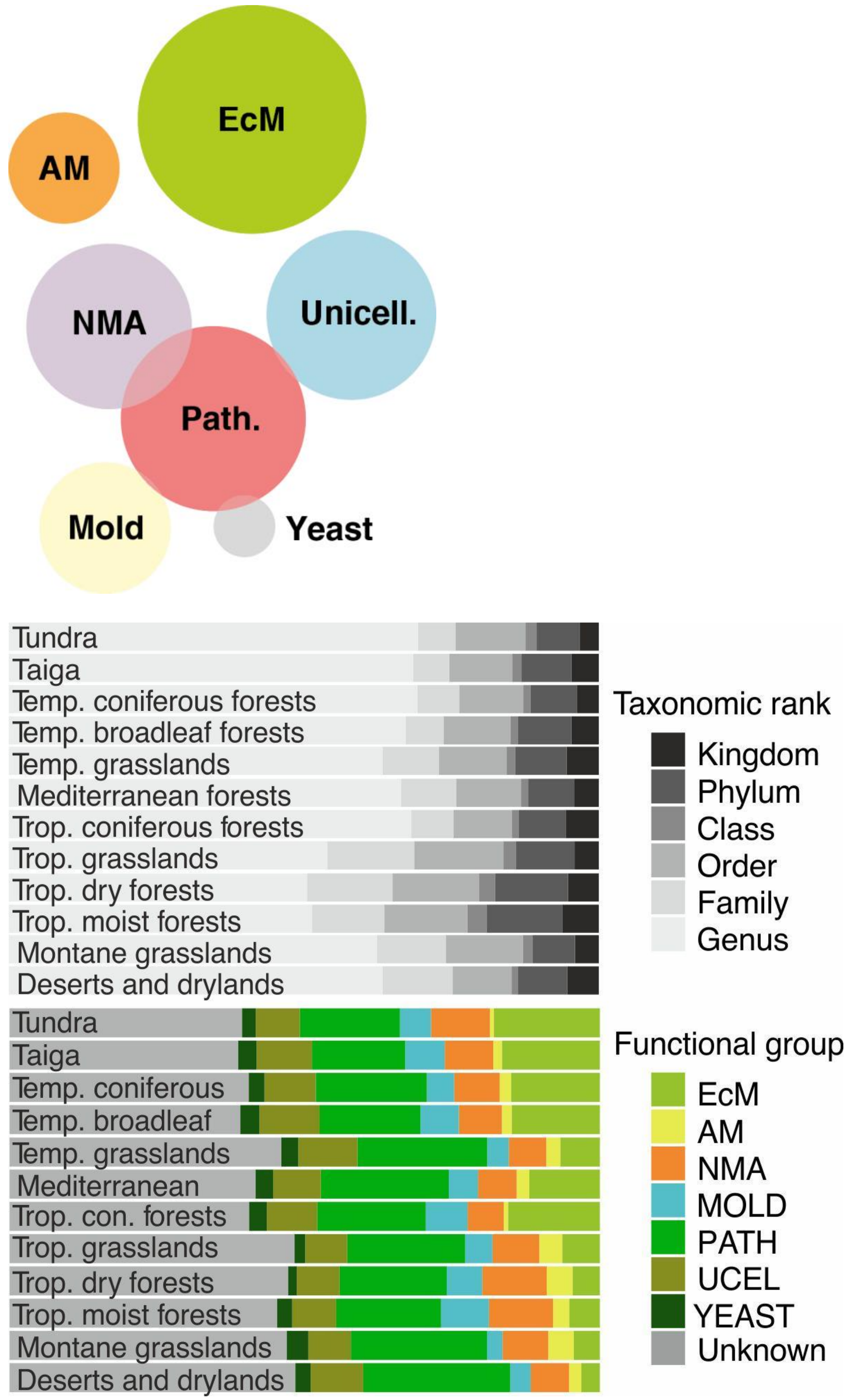

Fig. S3. Partitioning of OTU richness among fungal ecological groups.

(**A**) Partitioning of OTU richness among fungal ecological groups depicted as Euler diagram; AM, arbuscular mycorrhizal fungi; EcM, ectomycorrhizal fungi; NMA, non-ectomycorrhizal Agaricomycetes; Path., putative pathogens; Unicell., unicellular non-yeast fungi.

(**B**) Proportions of OTUs with the least resolved taxonomic annotation and functional annotation in biomes.

Alpha diversity

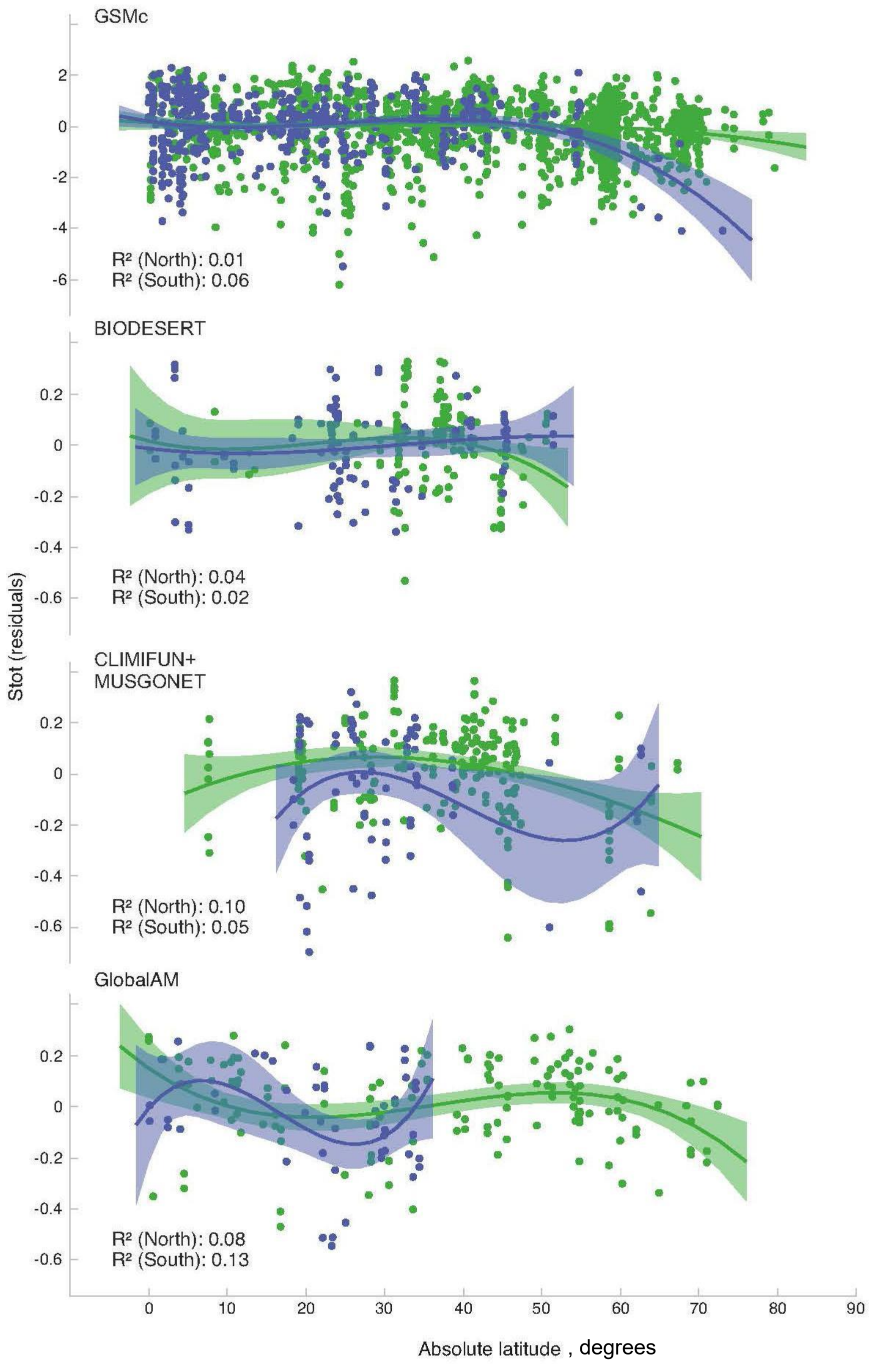

Fig. S4. Latitudinal distributions of OTU richness of all fungi (S_{TOT})

in the samples collected in different surveys in the northern hemisphere (green) and the southern hemispheres (blue). Coefficients of determination are shown for cubic polynomials.

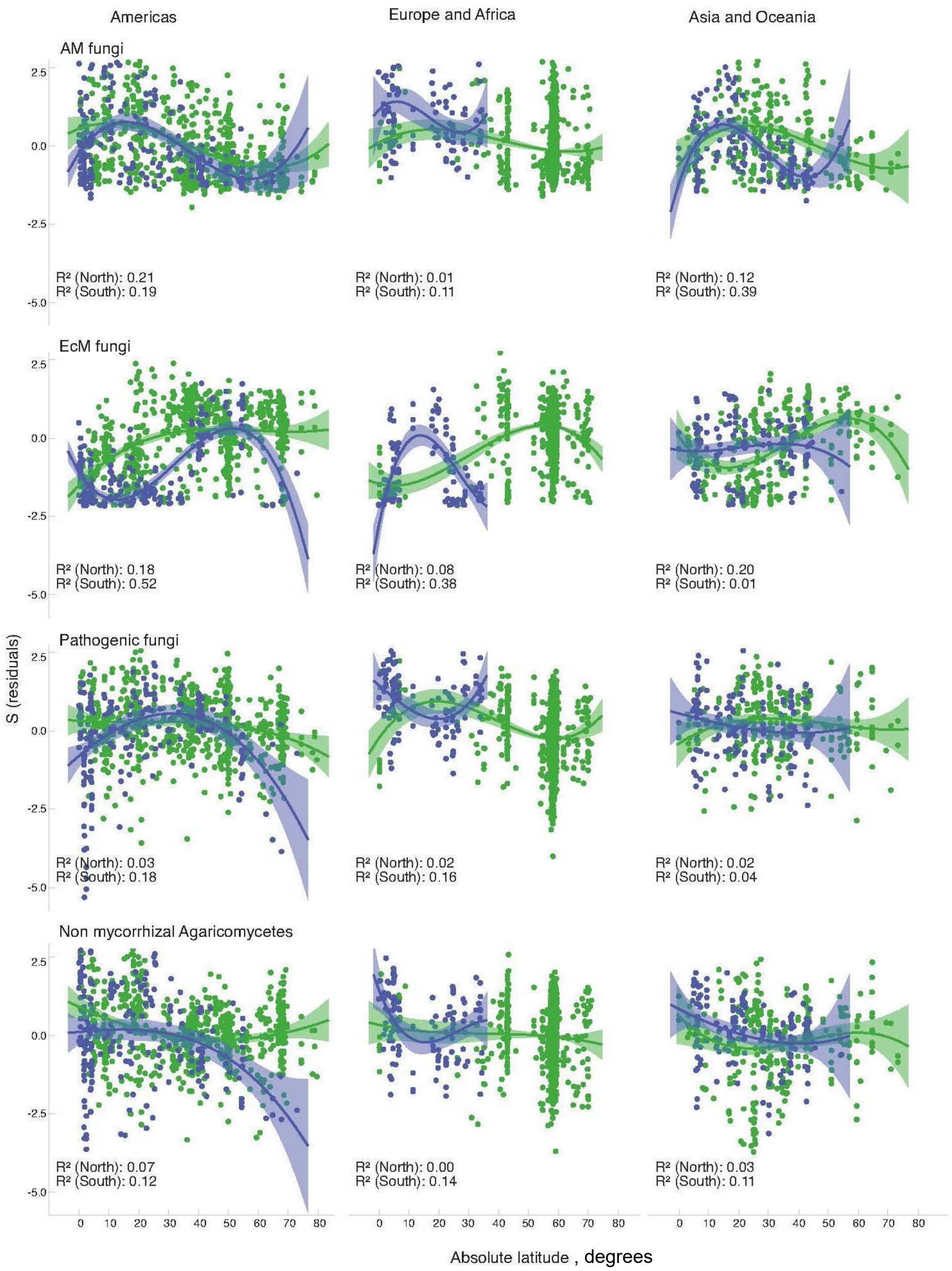

Fig. S4b. Latitudinal distribution of the alpha diversity (S) of fungal ecological groups.
The plot is based on different datasets in Northern (green) and Southern (blue) hemispheres. Coefficients of determination are shown for cubic polynomials.

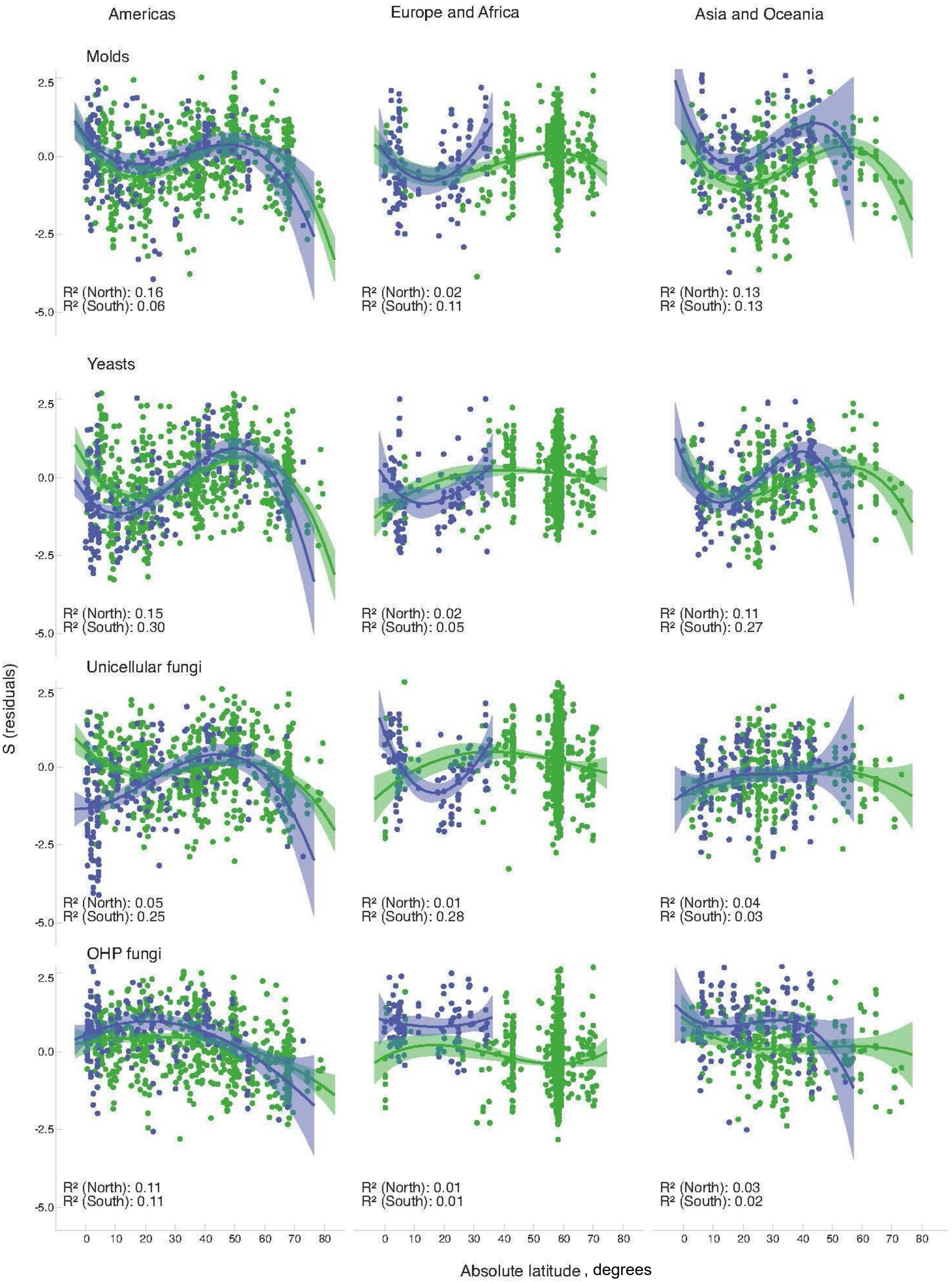

Fig. S4c. Latitudinal distribution of the alpha diversity (S) of fungal ecological groups based on different datasets in Northern (green) and Southern (blue) hemispheres. Coefficients of determination are shown for cubic polynomials.

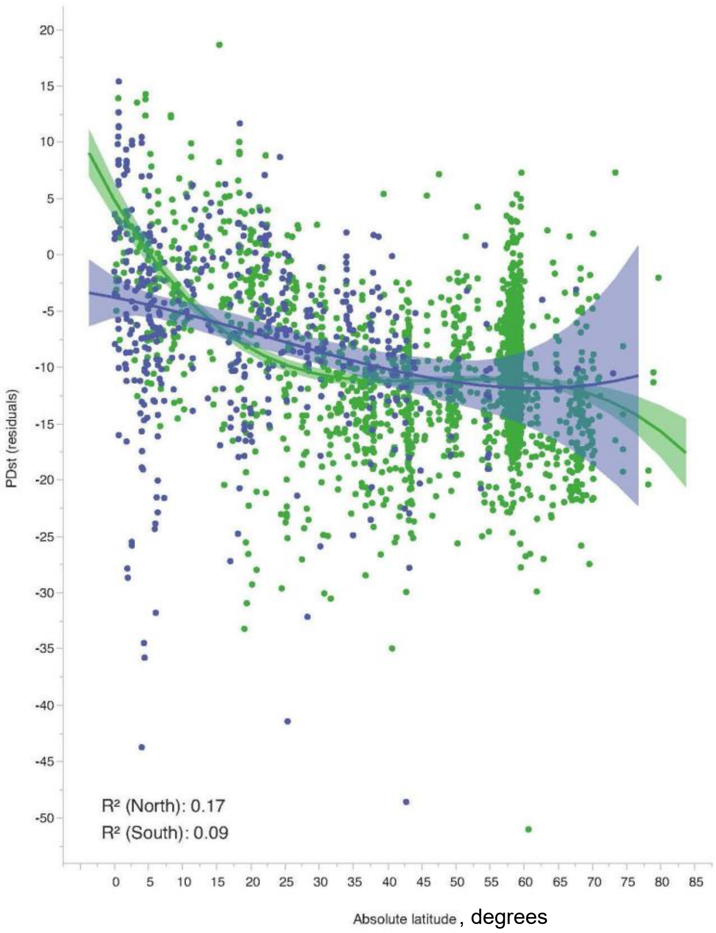

Fig. S4d. Latitudinal distributions of fungal phylogenetic alpha diversity (SES_{PD}) in GSMc dataset samples collected the northern hemisphere (green) and the southern hemispheres (blue). Coefficients of determination are shown for the cubic polynomials.

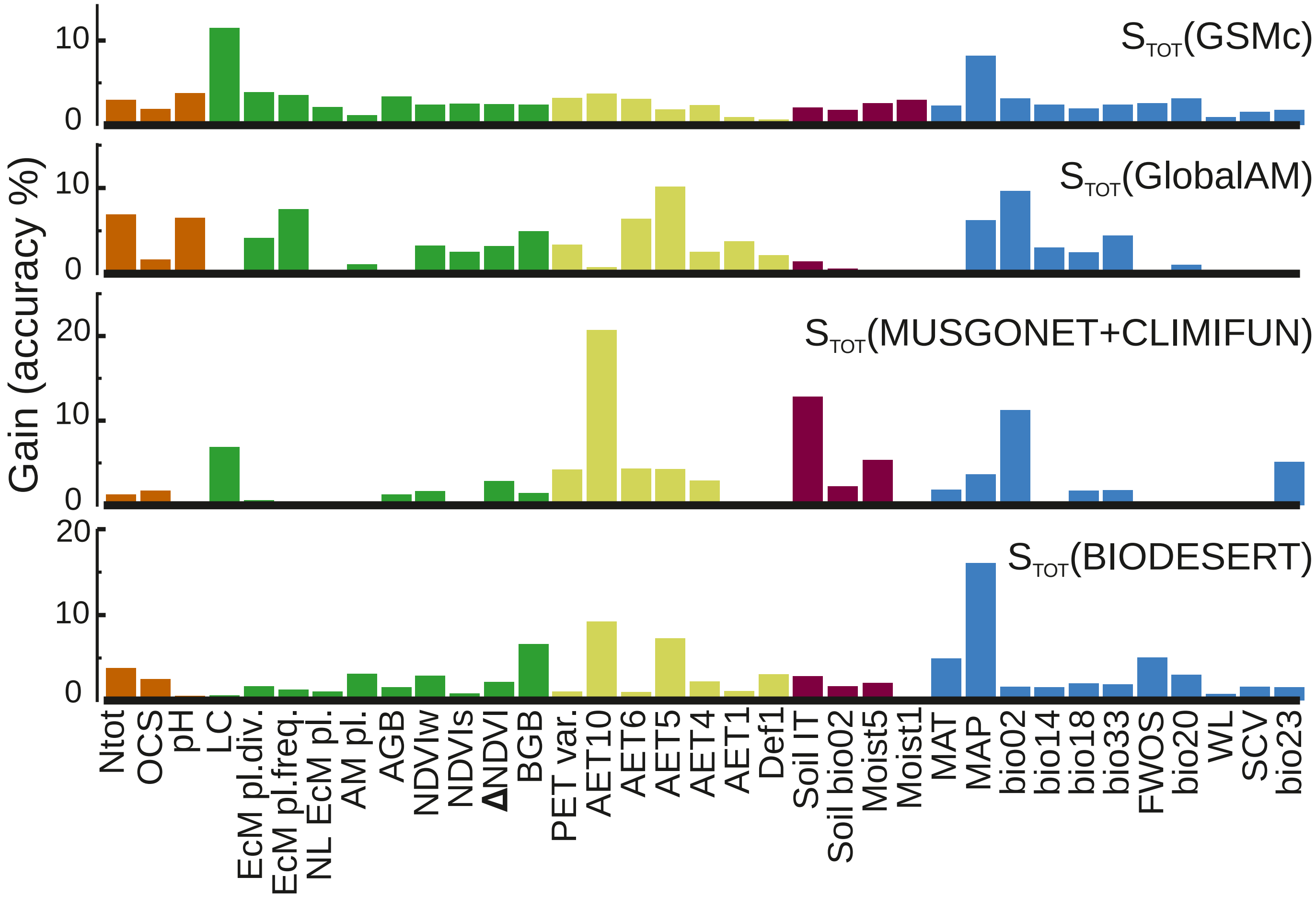

Fig. S5. Relative importance of environmental variables for S_{TOT} in different datasets.

Variables: edaphic (brown bars; OCS, organic carbon stock), coenotic (green bars; EcM pl. div., EcM pl. freq, and NL EcM pl. are the richness, frequency, and number of lineages of EcM plants, respectively; LC is land cover type; NDVI is normalized difference vegetation index in summer, winter, and its summer-winter difference; AGB is aboveground biomass carbon stock; BGB is belowground biomass carbon stock), coeno-climatic (yellow bars; PETvar is seasonality of potential evapotranspiration; AETx is monthly actual evapotranspiration; Def1 is moisture deficit in January), edapho-climatic (red bars; soil moisture, and soil climatic parameters (*59*)), and climatic (blue bars; bioclimatic parameters (*56*, *58*); MAT is mean annual temperature; MAP is mean annual precipitation; WL is winter length; FWOS is duration of snow-free frozen ground period; SCV is snow cover variability).

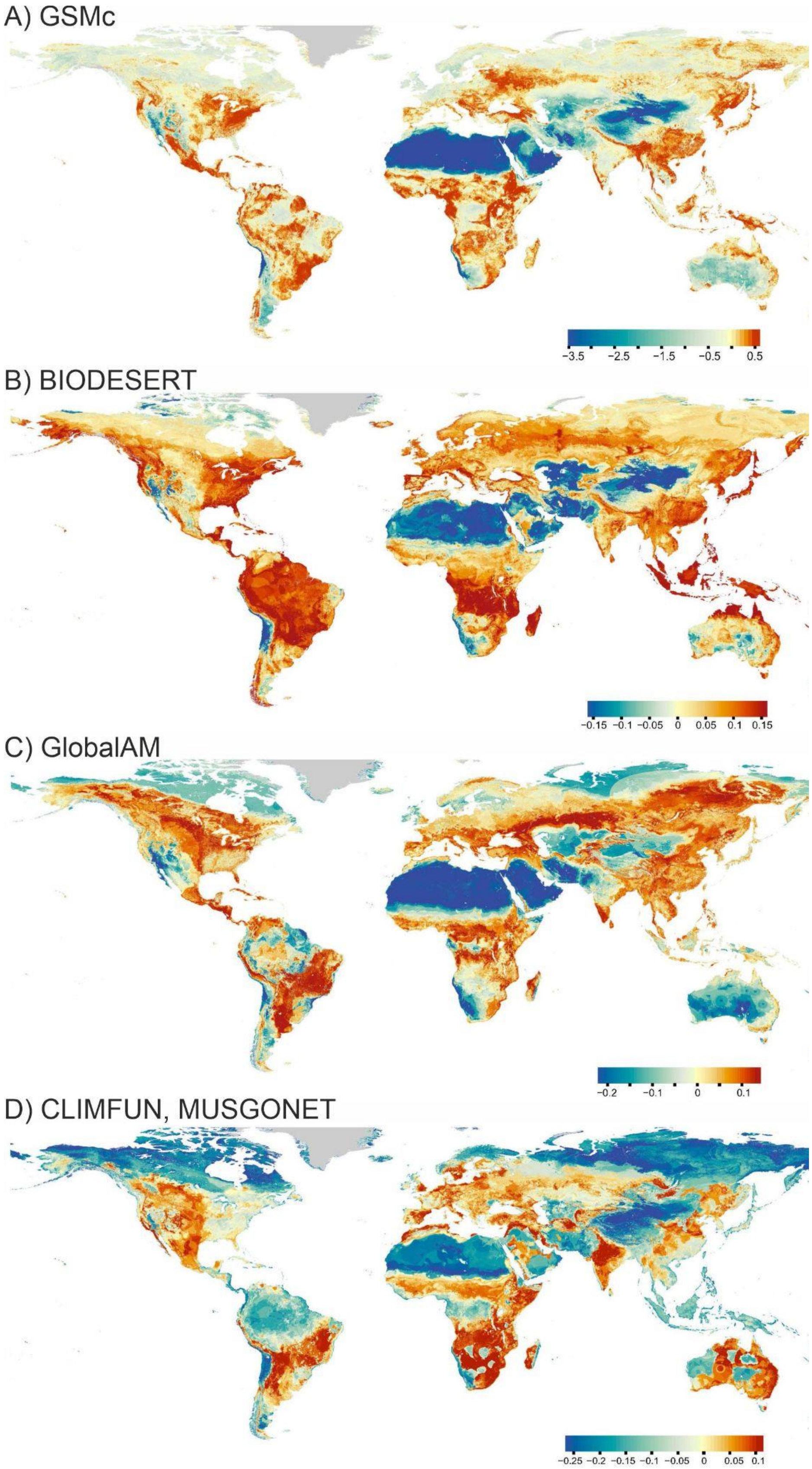

Fig. S6. Map of the expected fungal richness (S'_{TOT}) based on different datasets. (**A**) GSMc, (**B**) BIODESERT, (**C**) GlobalAM, and (**D**) CLIMFUN+MUSGONET.

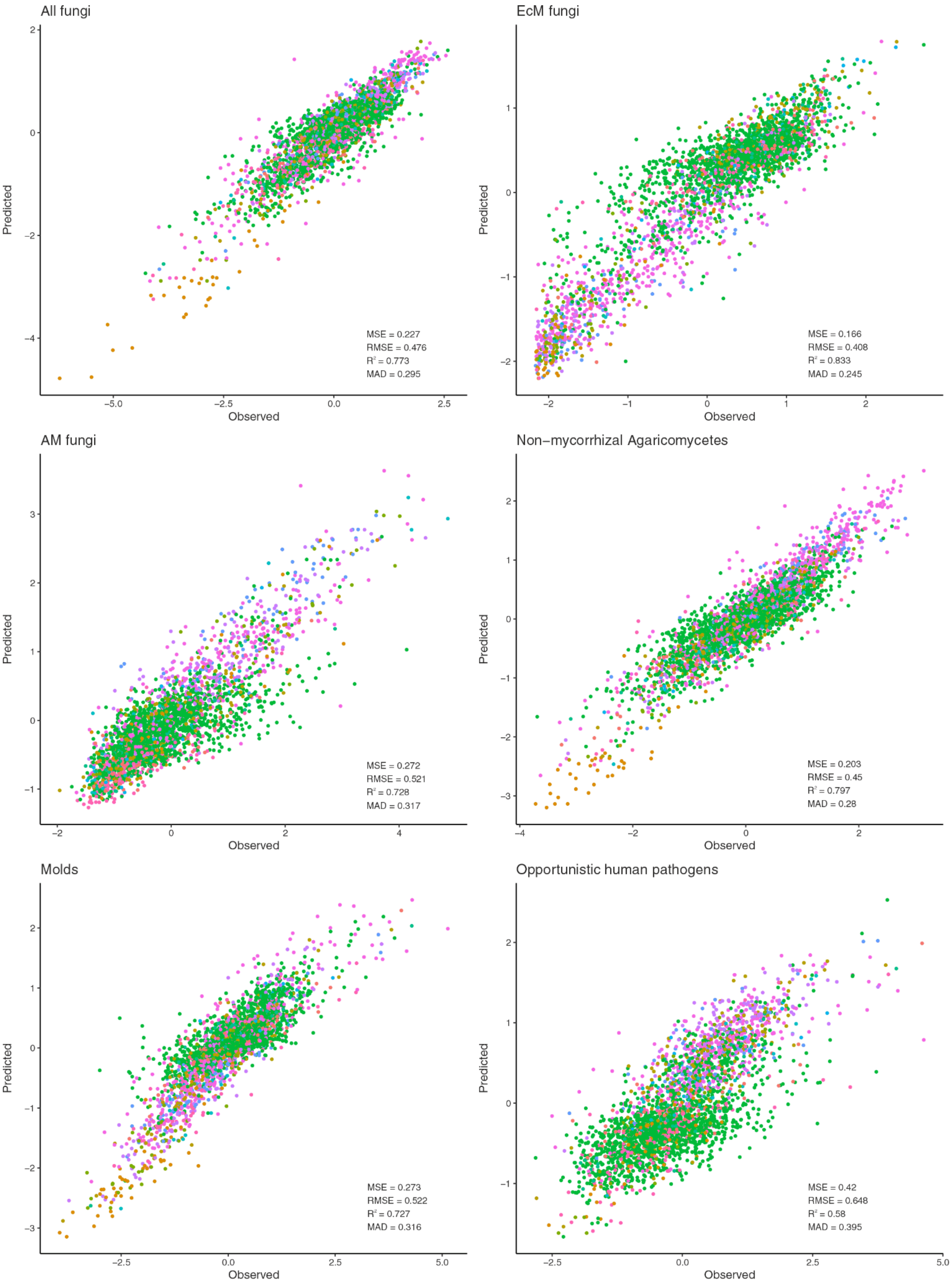

Fig. S7. Comparison of observed and predicted richness and phylogenetic diversity of fungi (based on residuals) obtained with XGBoost regression models.

Goodness-of-fit metrics displayed in the lower right corner (MSE and RMSE, mean-square error and root-mean-square error; MAD, mean absolute deviation). Color denotes biomes (see color legend below).

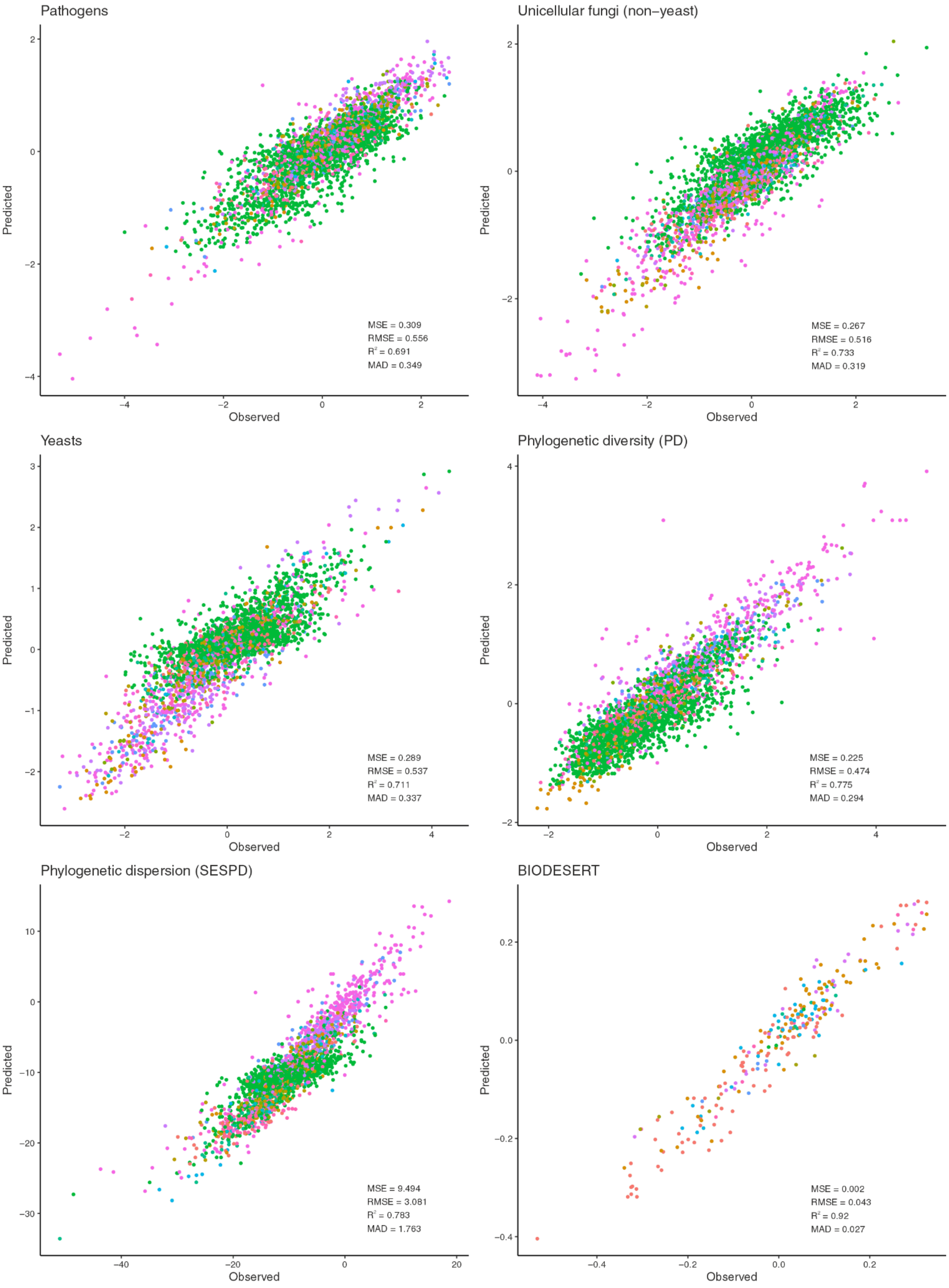

Fig. S7b. Comparison of observed and predicted richness and phylogenetic diversity of fungi.

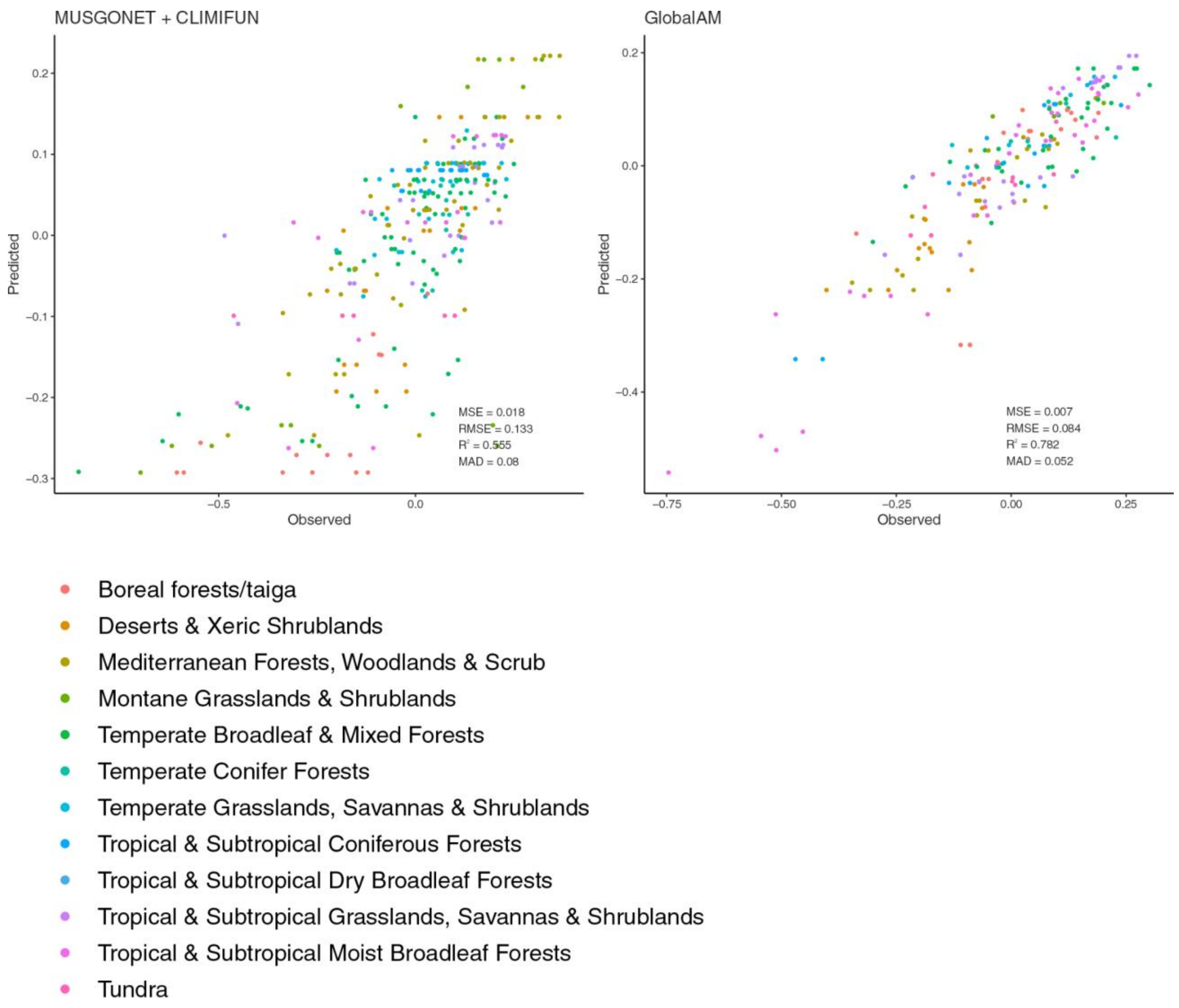

Fig. S7c. Comparison of observed and predicted richness and phylogenetic diversity of fungi.

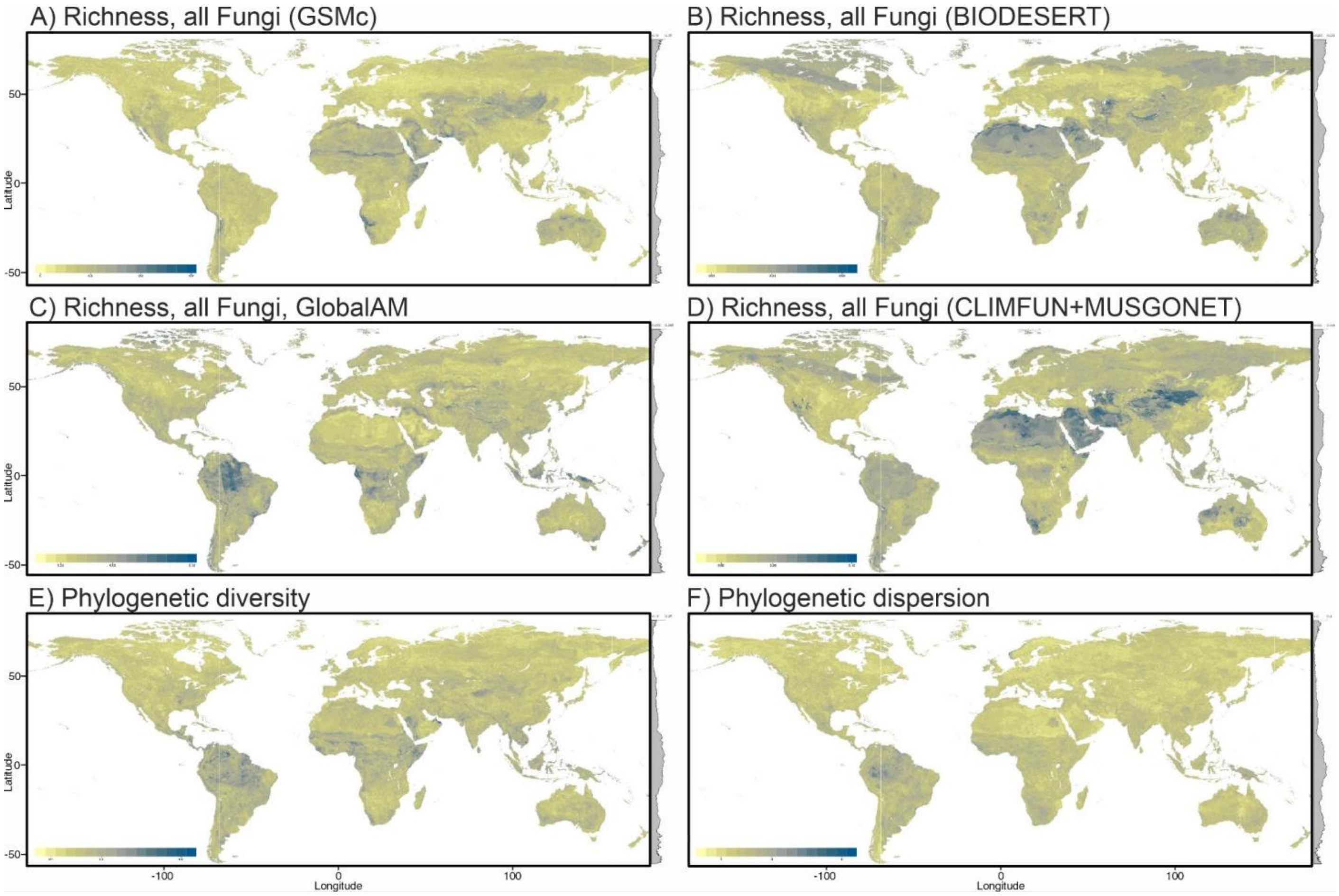

Fig. S8. The prediction uncertainty of fungal richness and phylogenetic diversity estimates.

The standard deviation of model predictions is used as a measure of uncertainty. Marginal distributions of the uncertainty values are shown as density plots to the right of the maps.

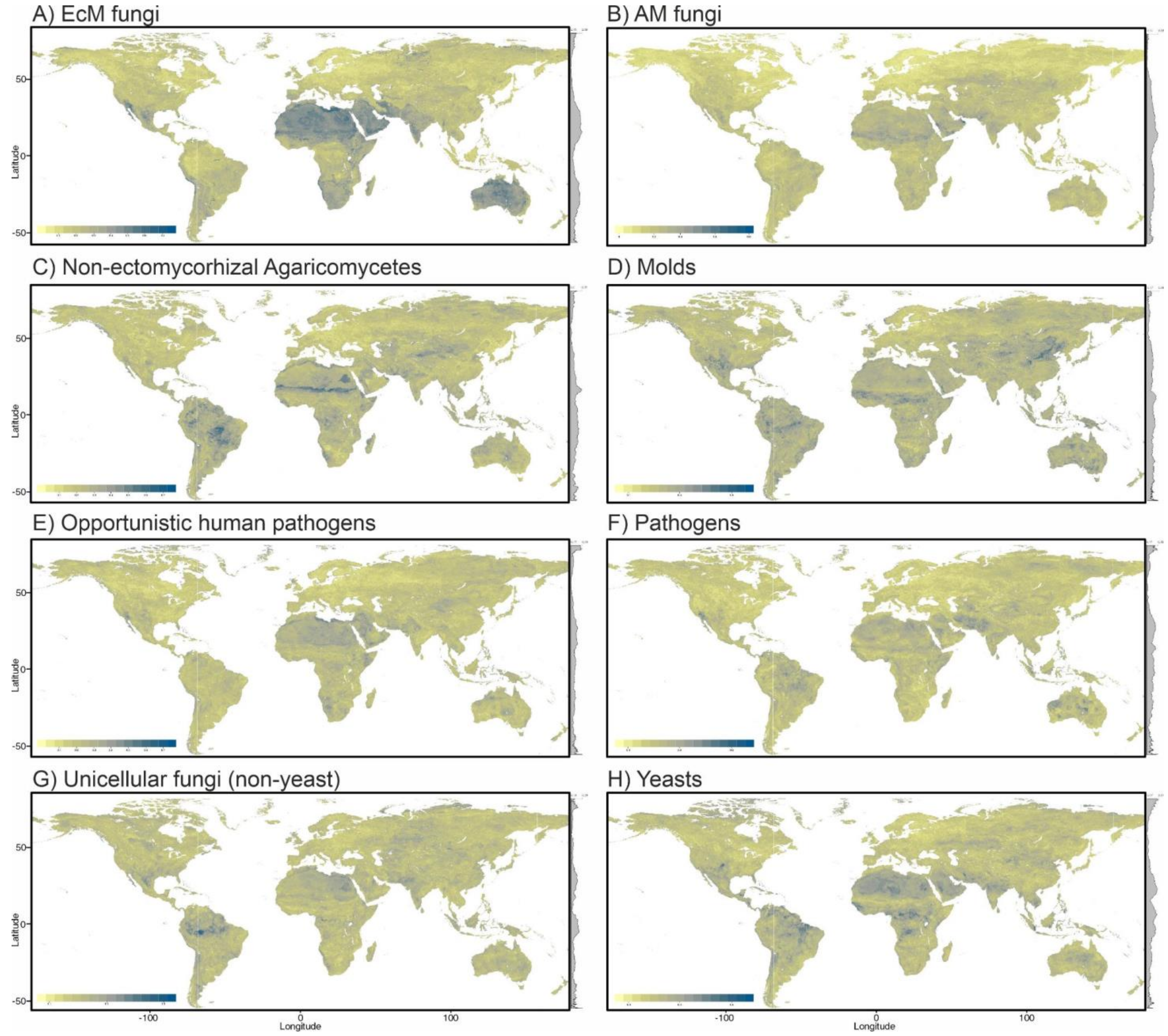

Fig. S8b. The prediction uncertainty of richness estimates for fungal ecological groups. The standard deviation of model predictions is used as a measure of uncertainty. Marginal distributions of the uncertainty values are shown as density plots to the right of the maps.

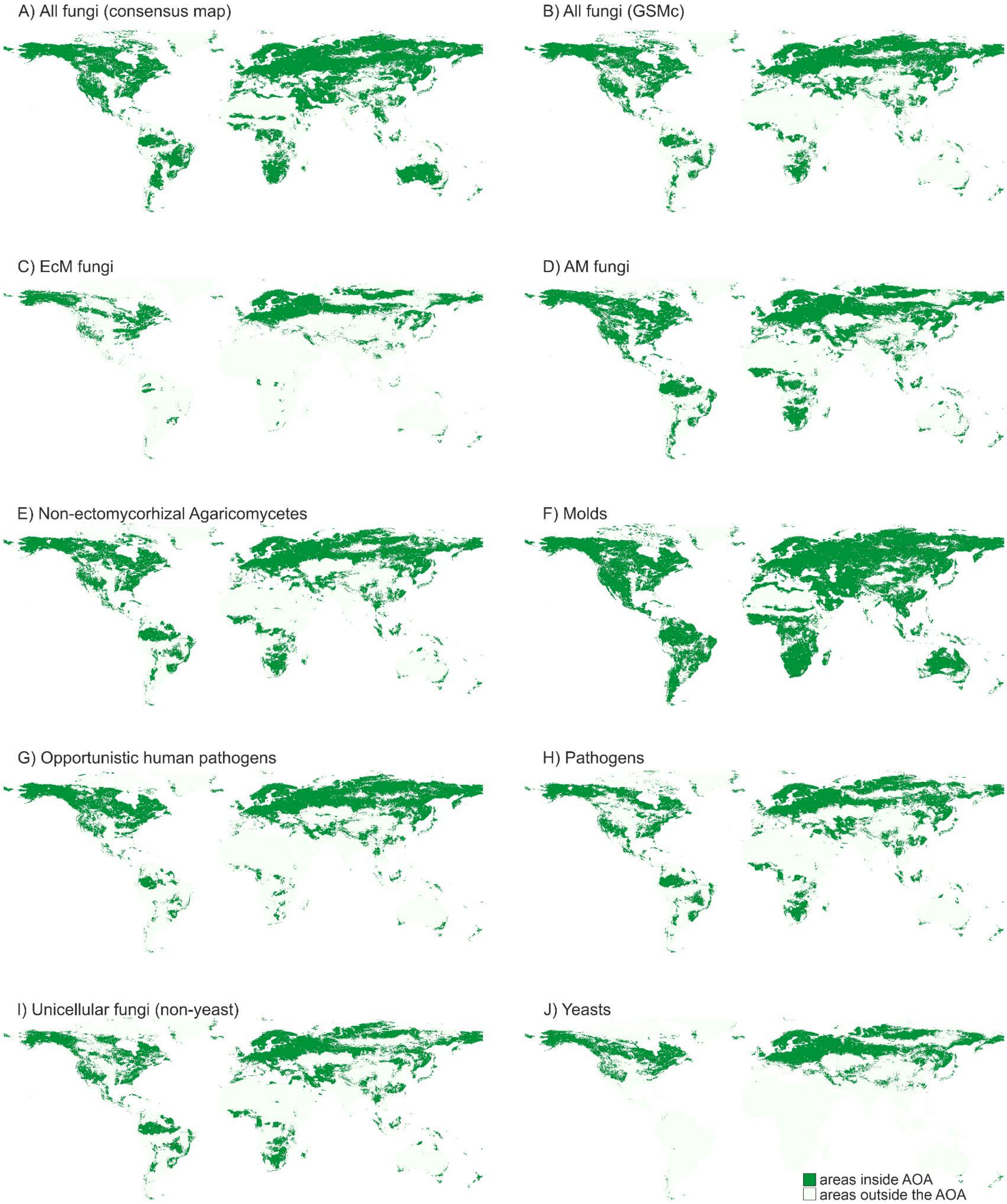

Fig. S9. Area of applicability (AOA) for the expected richness (S'_{TOT}) of fungi and fungal ecological groups.

Areas outside the AOA are shown in light-green and denote geographical space where environmental predictors differ from the values observed in the training data (considering the variable importance of predictors included in the model).

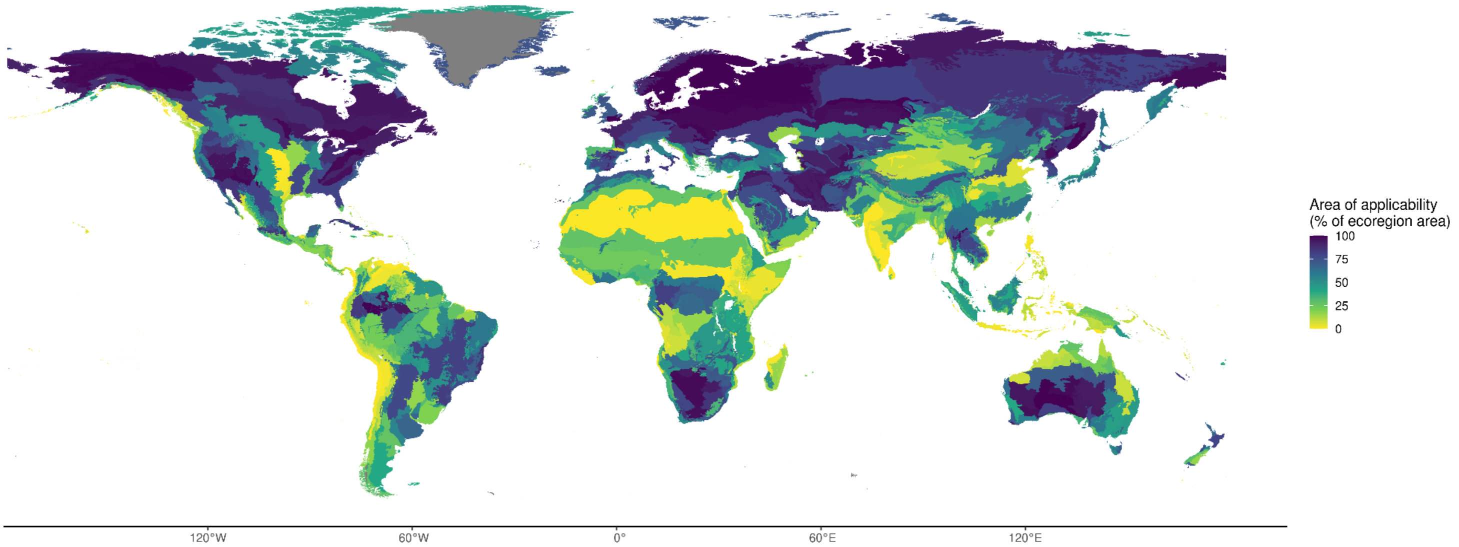

Fig. S9b. Ecoregion-scale estimates of the area of applicability (AOA), represented as a percentage of the ecoregion's total area.

A darker shade indicates a higher percentage of the area, suggesting that the samples in the training set aptly represent ecoregion's habitats. In contrast, lighter shades indicate smaller areas under AOA, pointing to regions with lower prediction precision. In particular, regions with pronounced environmental heterogeneity might necessitate a denser sampling to encompass all ecological niches, warranting future research emphasis. The grey color marks rock and ice areas, which were excluded from the analysis.

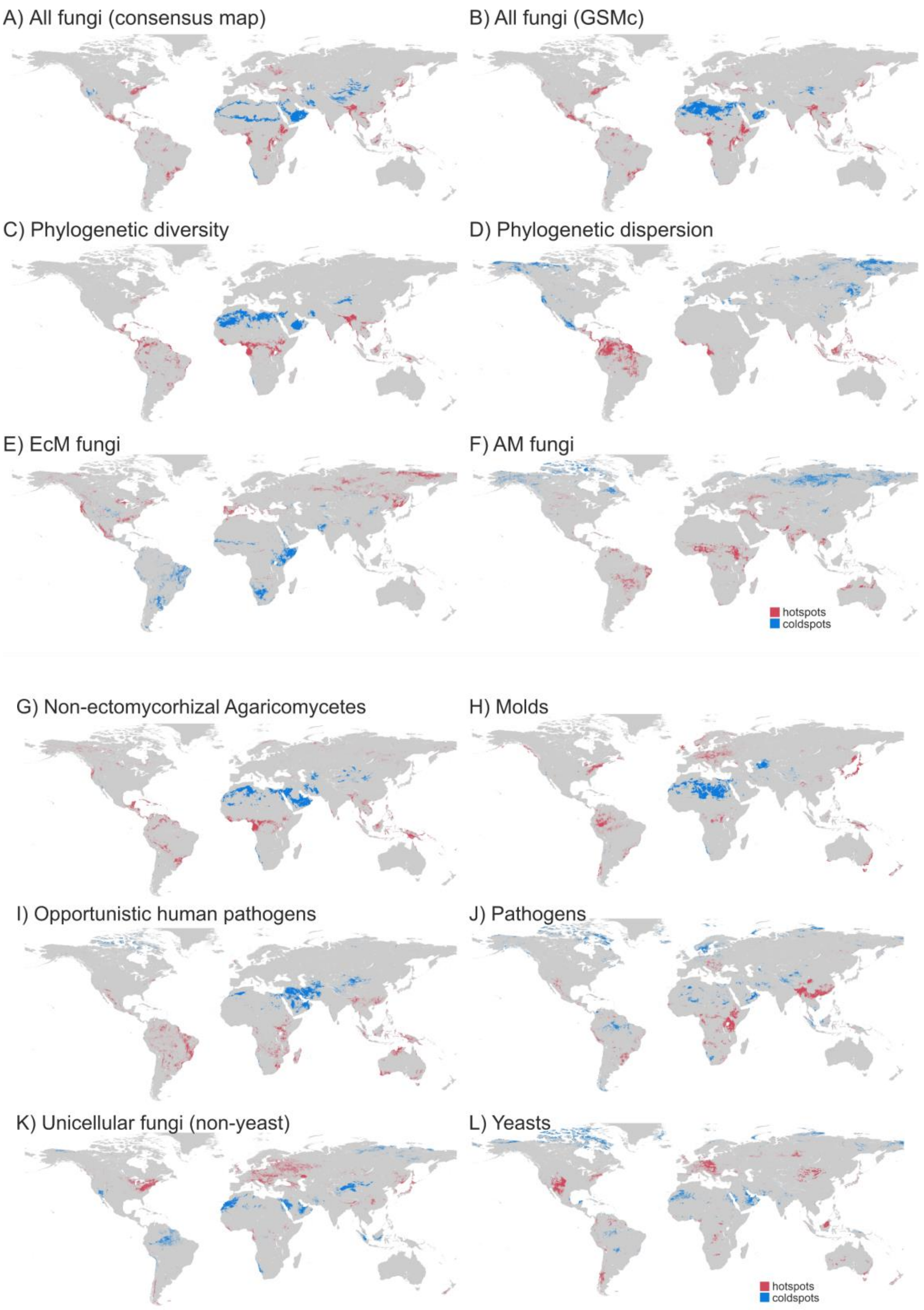

Fig. S10. Global distribution of hotspots and coldspots of fungal community diversity based on richness and phylogenetic diversity measures.

Red grid cells represent hotspots of diversity, indicating areas with the highest 2.5% percentile of global diversity, while blue grid cells represent coldspots of diversity, indicating areas with the lowest 2.5% percentile of diversity.

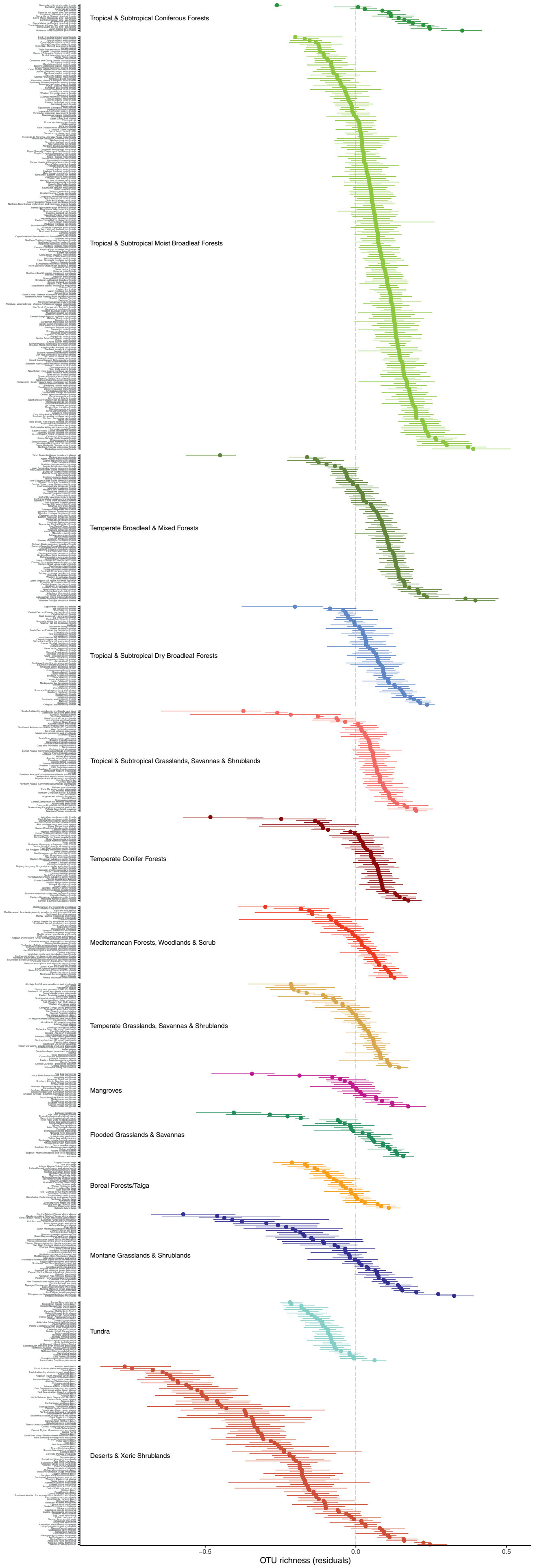

Fig. S11. Ecoregional S′$_{TOT}$ (median ± IQR).

Ecoregions are ranked within biomes by S’$_{TOT}$. Dashed vertical line is the global median S’$_{TOT}$.

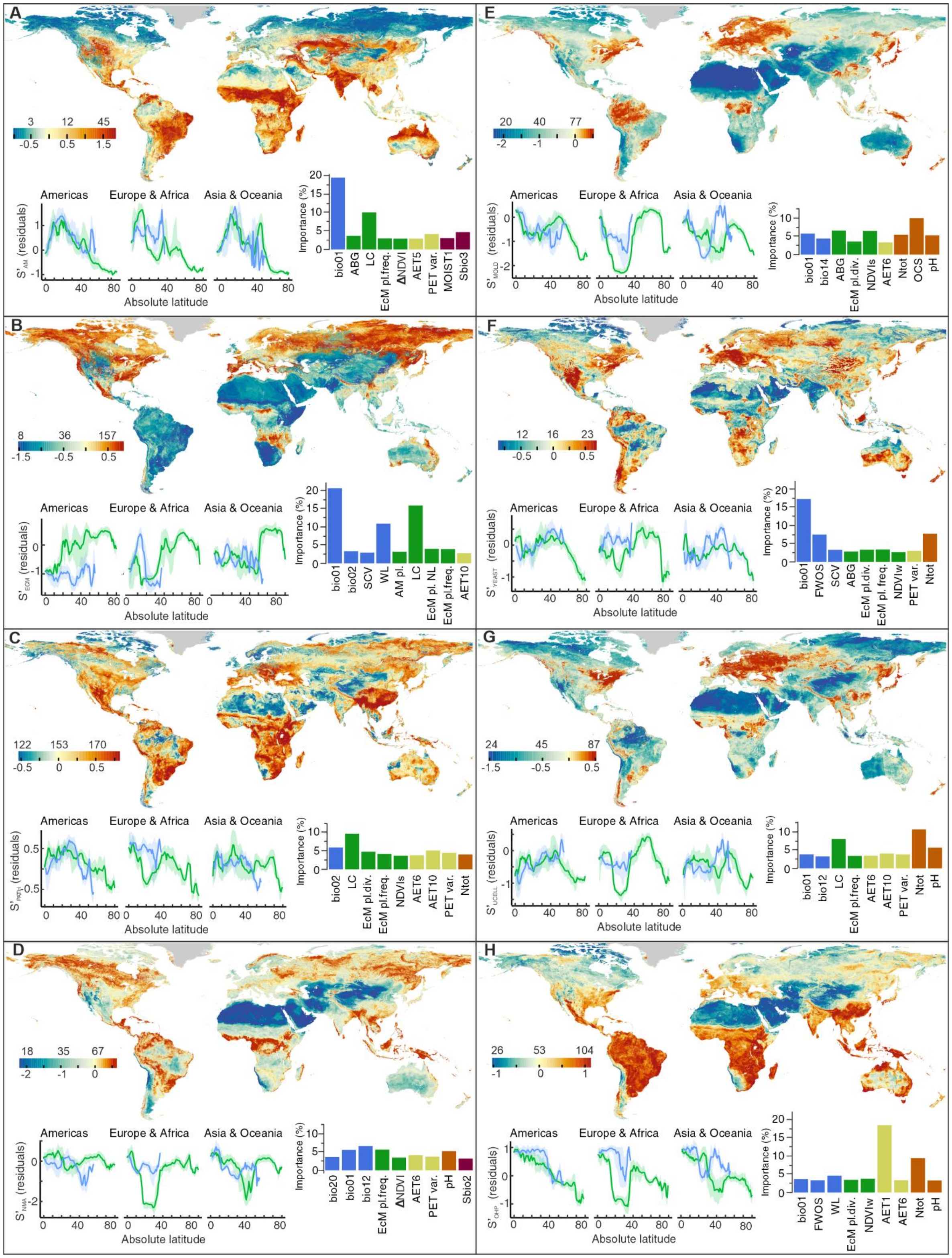

Fig. S12. Global distribution of the alpha diversity of fungal ecological groups in soil.

Predicted richness (S′) of (**A**) AM fungi, (**B**) EcM fungi, (**C**) pathogens, (**D**) non-mycorrhizal Agaricomycetes, (**E**) molds, (**F**) yeasts, (**G**) unicellular fungi, and (**H**) opportunistic human pathogens. Above and below the color legends, respectively, OTU numbers and residuals are shown. Plots show latitudinal distributions of S′ for each ecological group through Americas (left), Europe and Africa (central), and Asia and Oceania (right). Blue and green lines denote southern and northern hemispheres, respectively. Barplots show the importance of the nine most influential environmental variables for the group's S (for variable names, see Fig. S5 footnote).

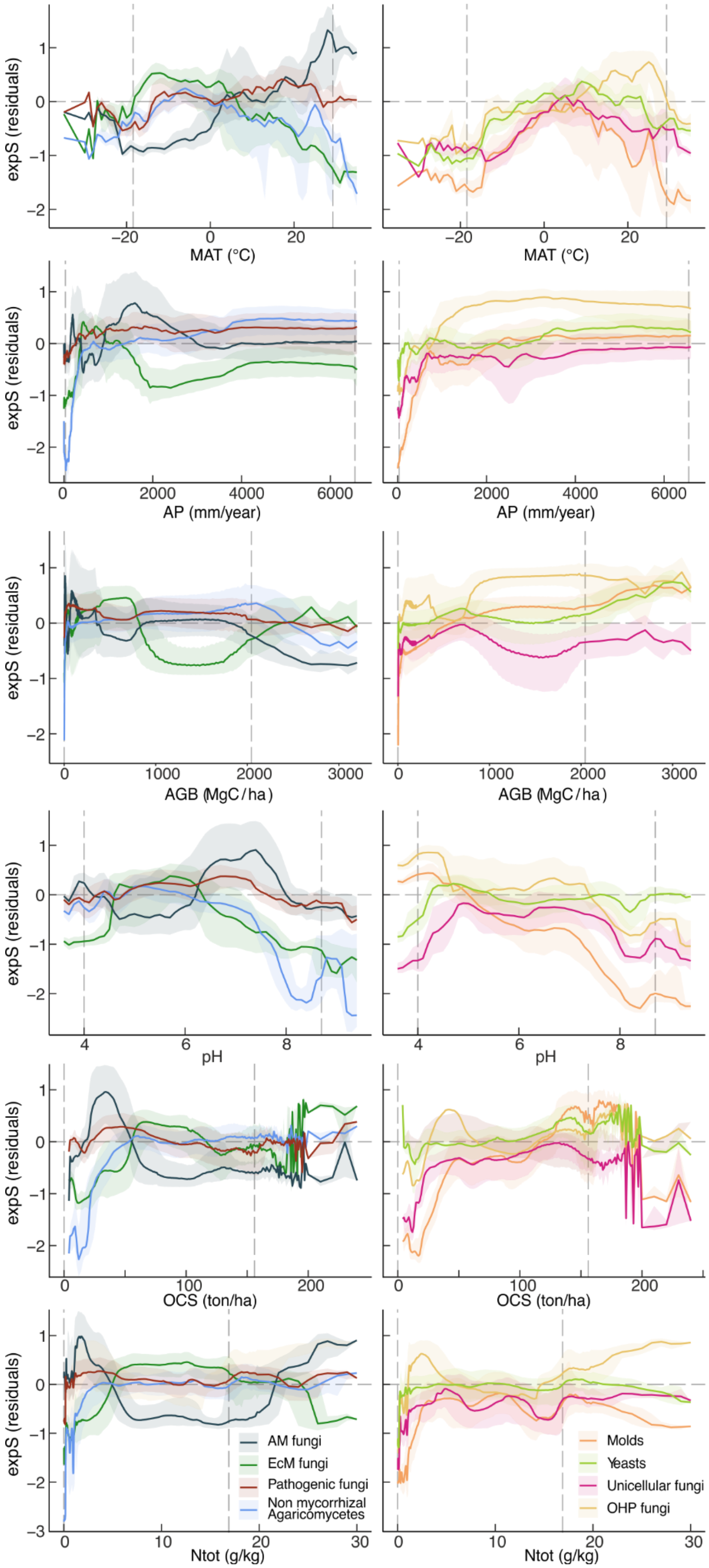

Fig. S13. Relationships of the richness of ecological groups with environmental variables.

Distribution of S′ of fungal ecological groups along the gradients of mean annual temperature (MAT), mean annual precipitation (AP), aboveground biomass carbon stock (AGB), soil pH, soil organic carbon stock (OCS), and soil total nitrogen content (Ntot). Vertical dashed lines denote the within-AOA margins of the variable range.

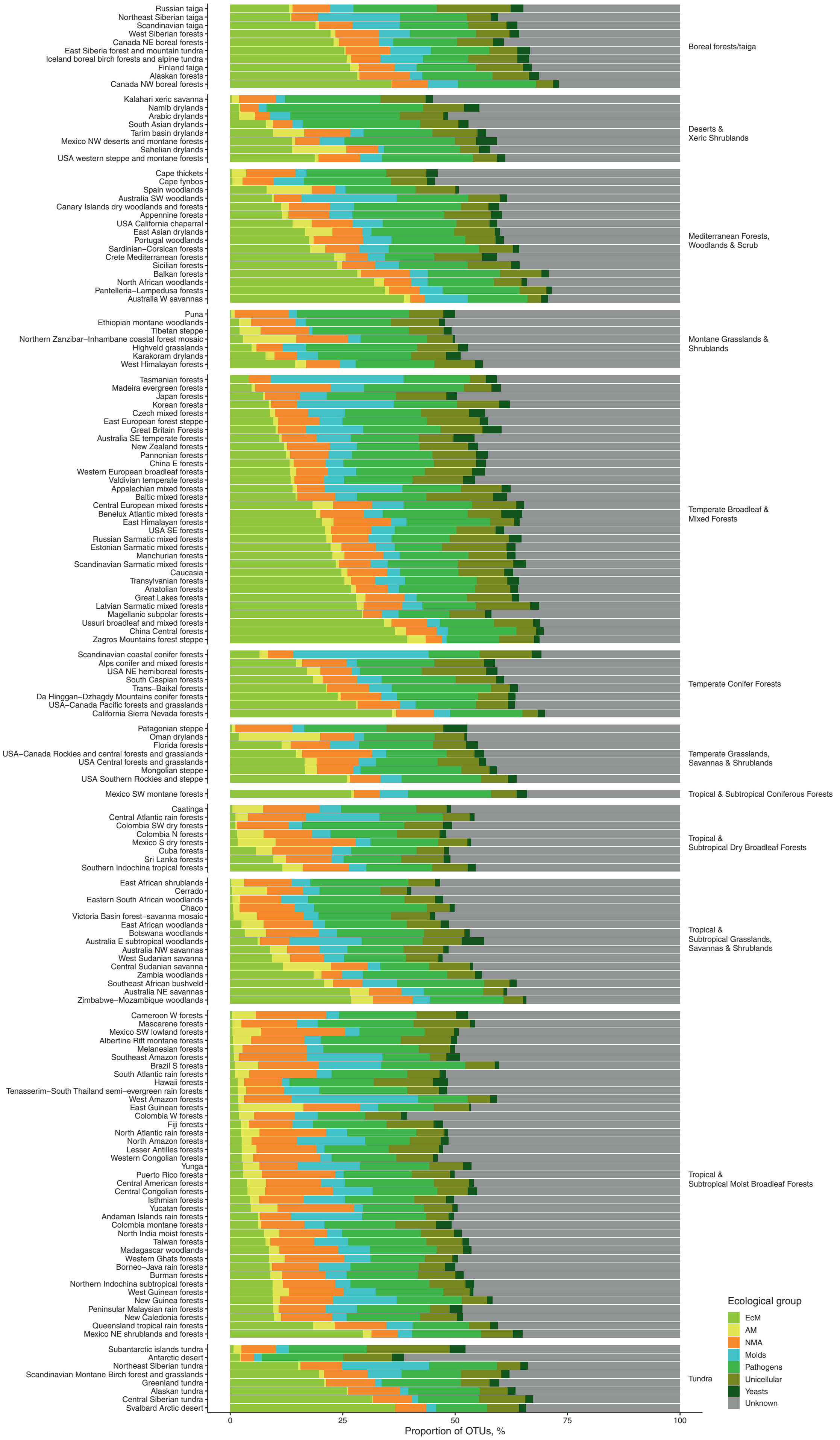

Fig. S14. Proportions of fungal ecological groups across ecoregions.

Based on observed data.

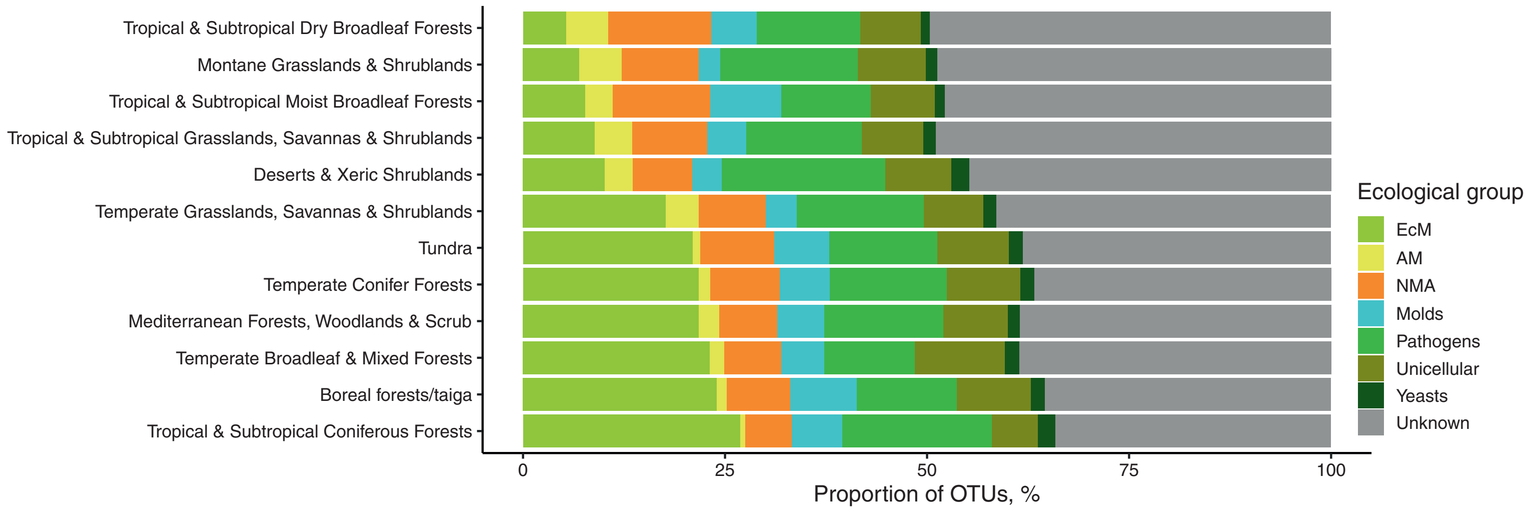

Fig. S15. Proportions of fungal ecological groups across biomes.

Based on observed data.

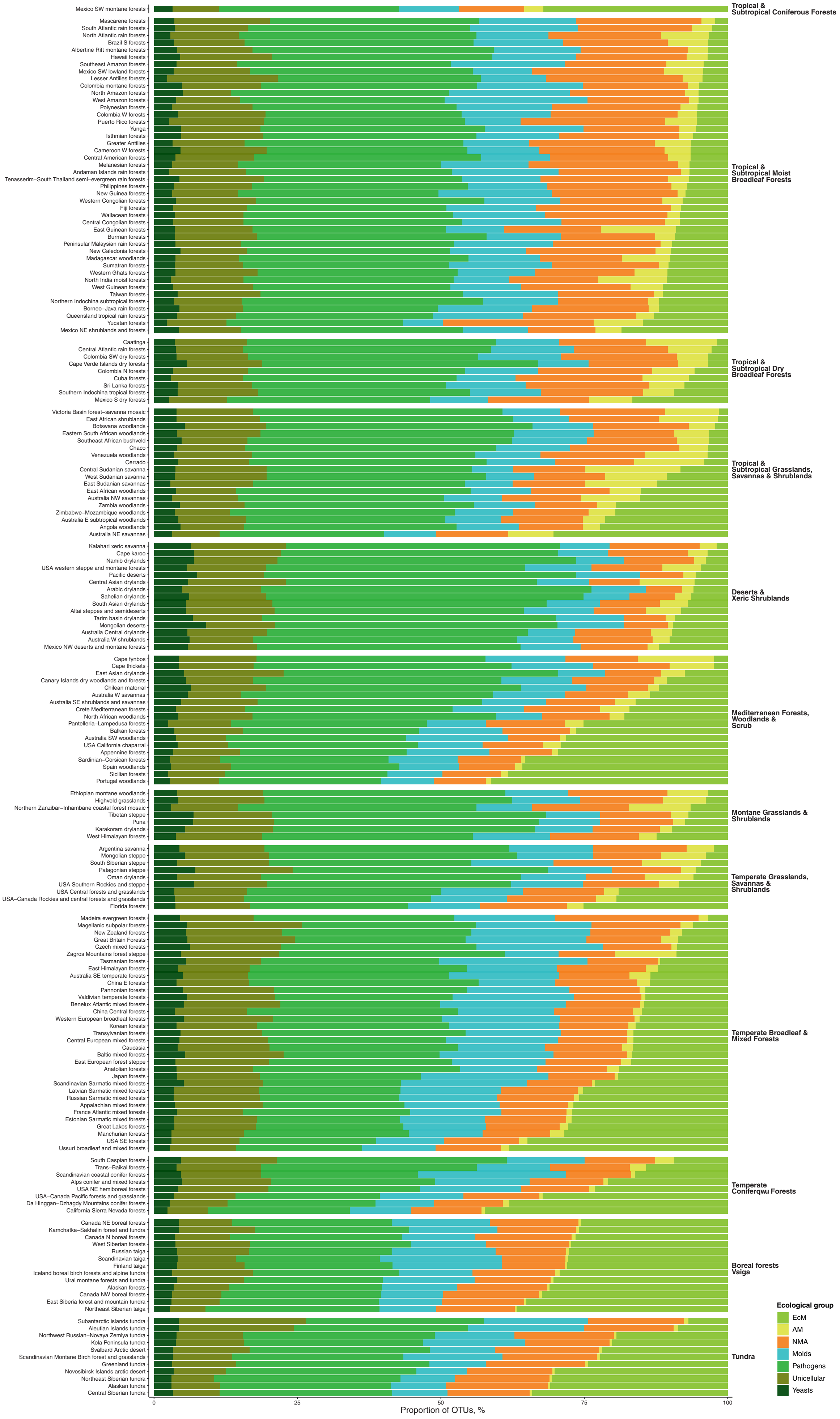

Fig. S16. Predicted profiles of fungal ecological groups across ecoregions.

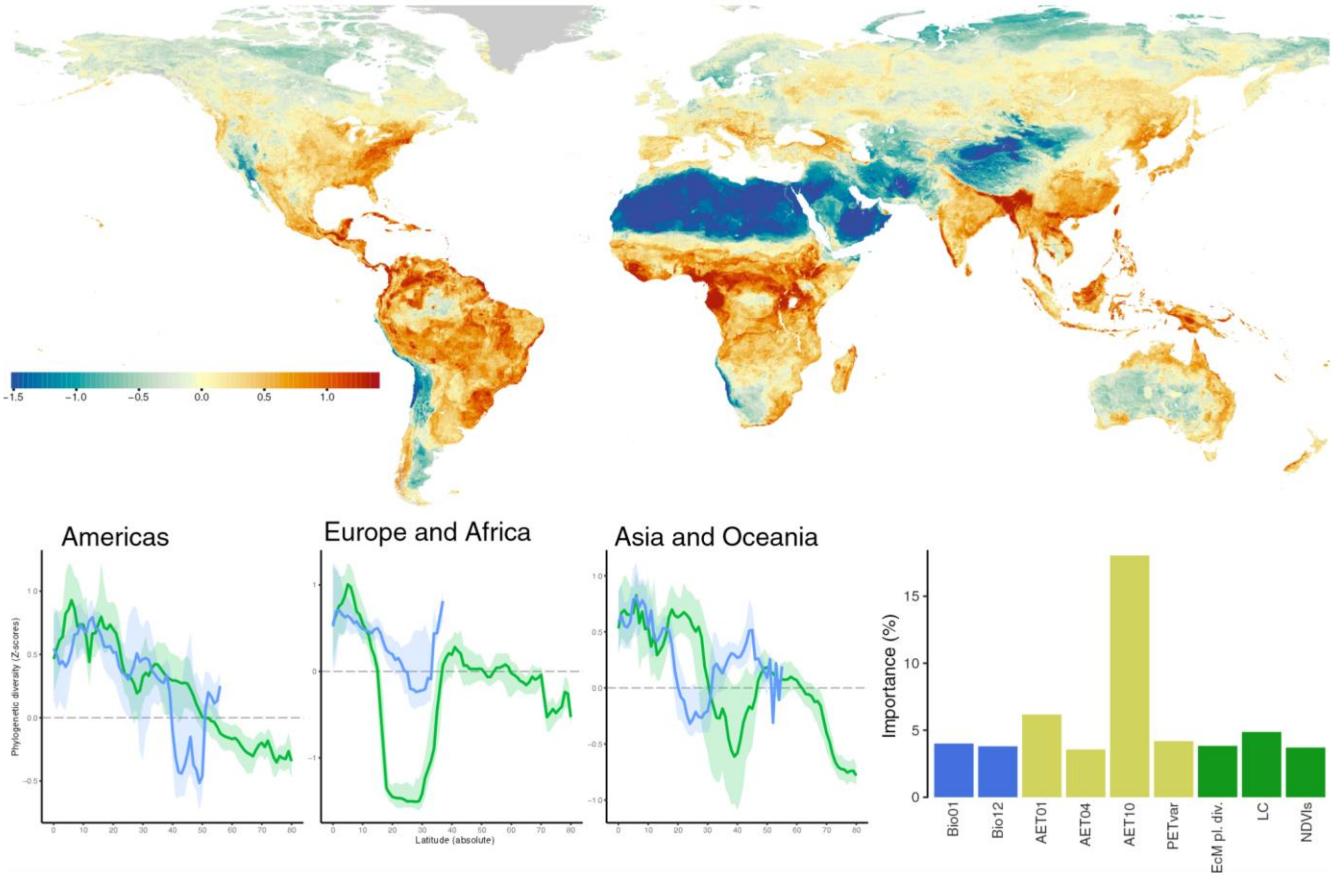

Fig. S17. Predicted global distribution of phylogenetic alpha diversity (S_{PD}') of soil fungi.

Below the map, latitudinal distribution of the indices in Northern (green) and Southern (blue) hemispheres (left, Americas; middle, Europe and Africa; right, Asia and Oceania) as well as the importance of the most influential environmental variables are shown.

Beta Diversity

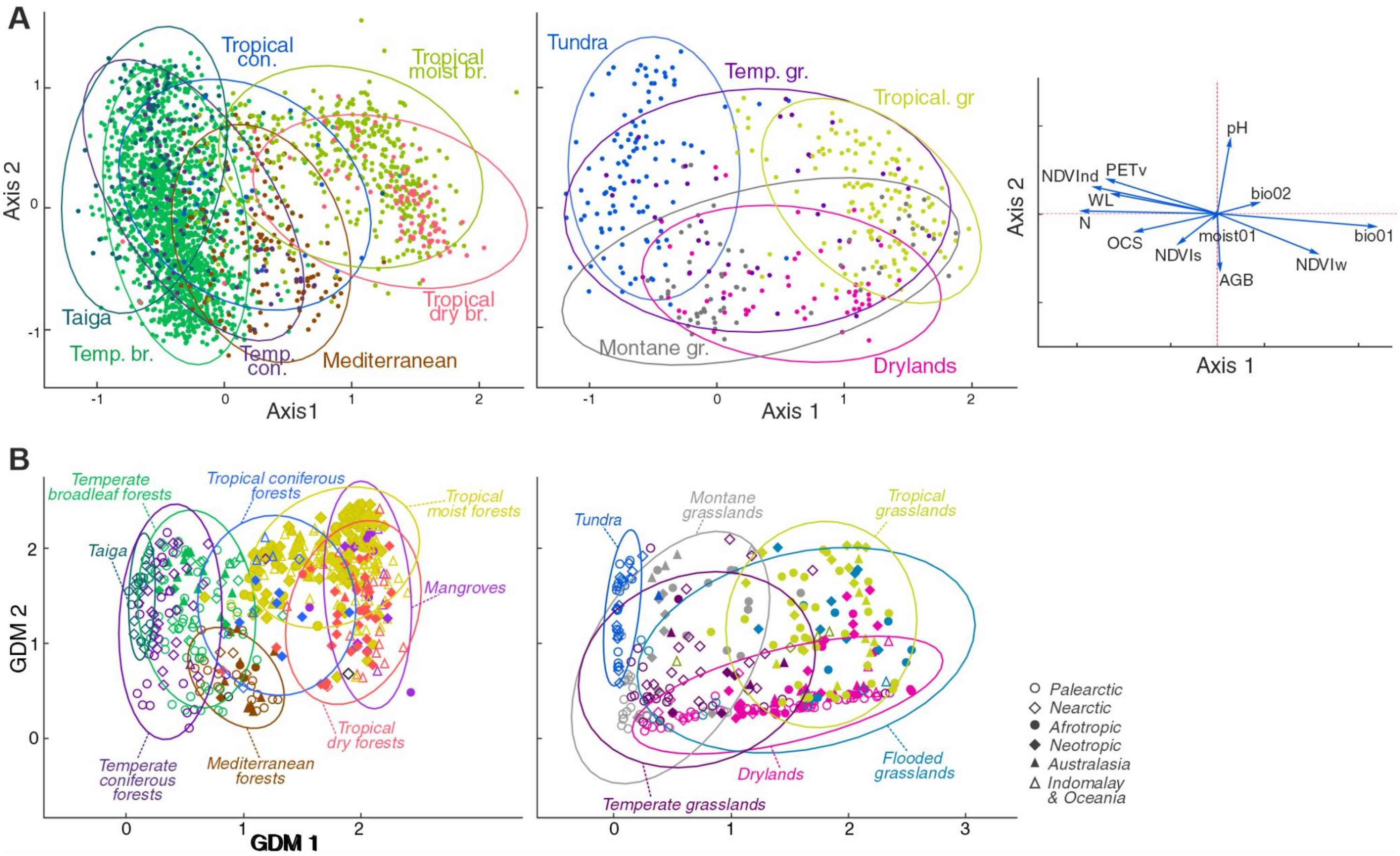

Fig. S18. Compositional similarity of soil fungal communities.

(**A**) PCoA ordination of GSMc samples by OTU composition. Each biome type is highlighted by a specific color. Diagrams indicate biomes with a dominance of woody vegetation (located on the left), biomes characterized by herbaceous vegetation (middle), and contribution of predictor variables to ordination axes (right). Con. denotes coniferous forests; br., broadleaf forests; gr., grasslands, savannas, and shrublands.

(**B**) GDM-based ordination of ecoregion centroids (based on D_{TAX} values; Fig. 4D). Biome types are color-coded, and the point shape denotes biogeographic realm. The predicted OTU composition along the first axis demonstrates a strong correlation with climate temperature (r_{MAT} = 0.90), soil N content (r = 0.77), and seasonality parameters (r >= |0.70|); the second and third axes are correlated with soil pH (r = 0.58) and soil temperature diurnal range (r = -0.55), respectively.

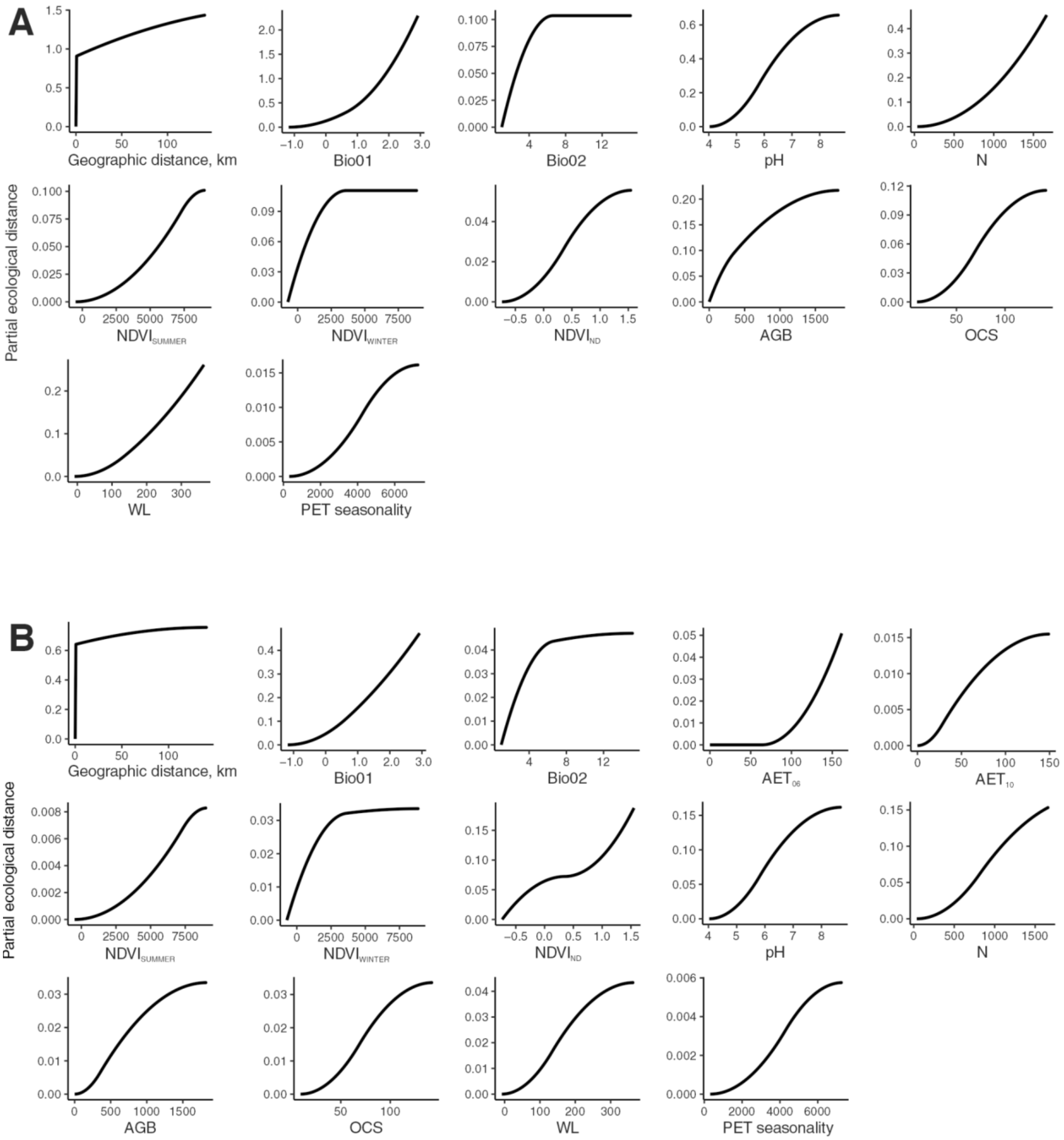

Fig. S19. Fitted I-splines of environmental variables affecting.

(**A)** OTU (D_{TAX}) and **(B)** phylogenetic lineages (D_{PD}) composition of soil fungal communities as identified by the generalized dissimilarity modeling (GDM). The relative y-axis range of variables indicates the relative strength of that variable in determining community dissimilarity, while the nonlinearity of the response indicates which sections of the environmental gradient have steeper predicted compositional dissimilarity. Bio01, mean annual temperature (℃); Bio02, mean diurnal range (℃); AET, actual monthly evapotranspiration (mm); AGB, aboveground biomass carbon density (MgC/ha); N, total nitrogen in soil (cg/kg); NDVI, normalized difference vegetation index; $NDVI_{ND}$, normalized difference between summer and winter NDVI; OCS, organic carbon stocks (ton/ha); PET seasonality, monthly variability in potential evapotranspiration (mm/month); WL, winter length (number of days)

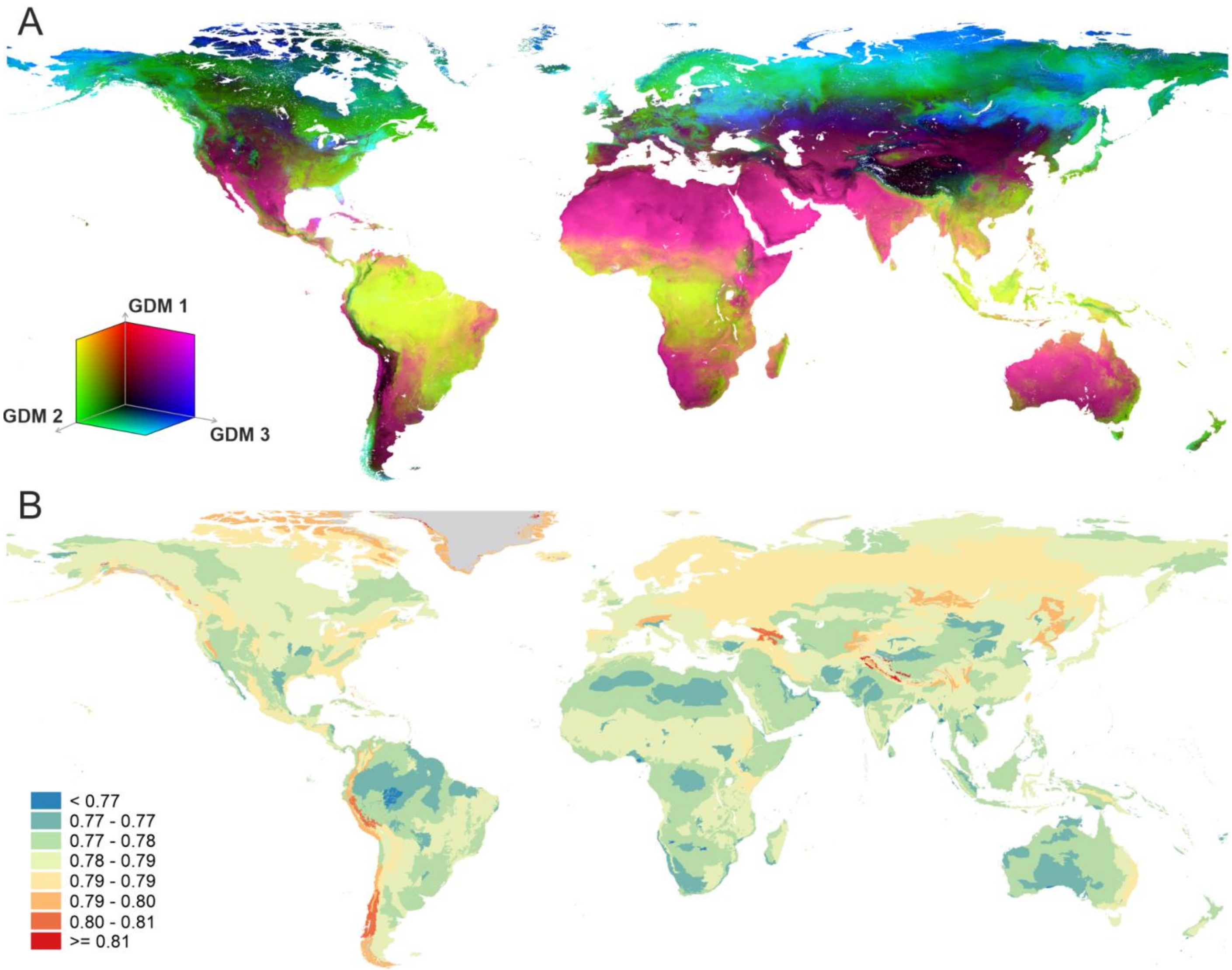

Fig. S20. Predicted phylogenetic dissimilarity of soil fungal communities.

(**A**) Gradients in phylogenetic lineages composition derived from GDM-transformed environmental predictors. Color similarity between localocations is proportional to phylogenetic similarity of their fungal communities. (**B**) Predicted dissimilarity in phylogenetic lineages composition among soil fungal communities within ecoregions (median of D'_{PD}).

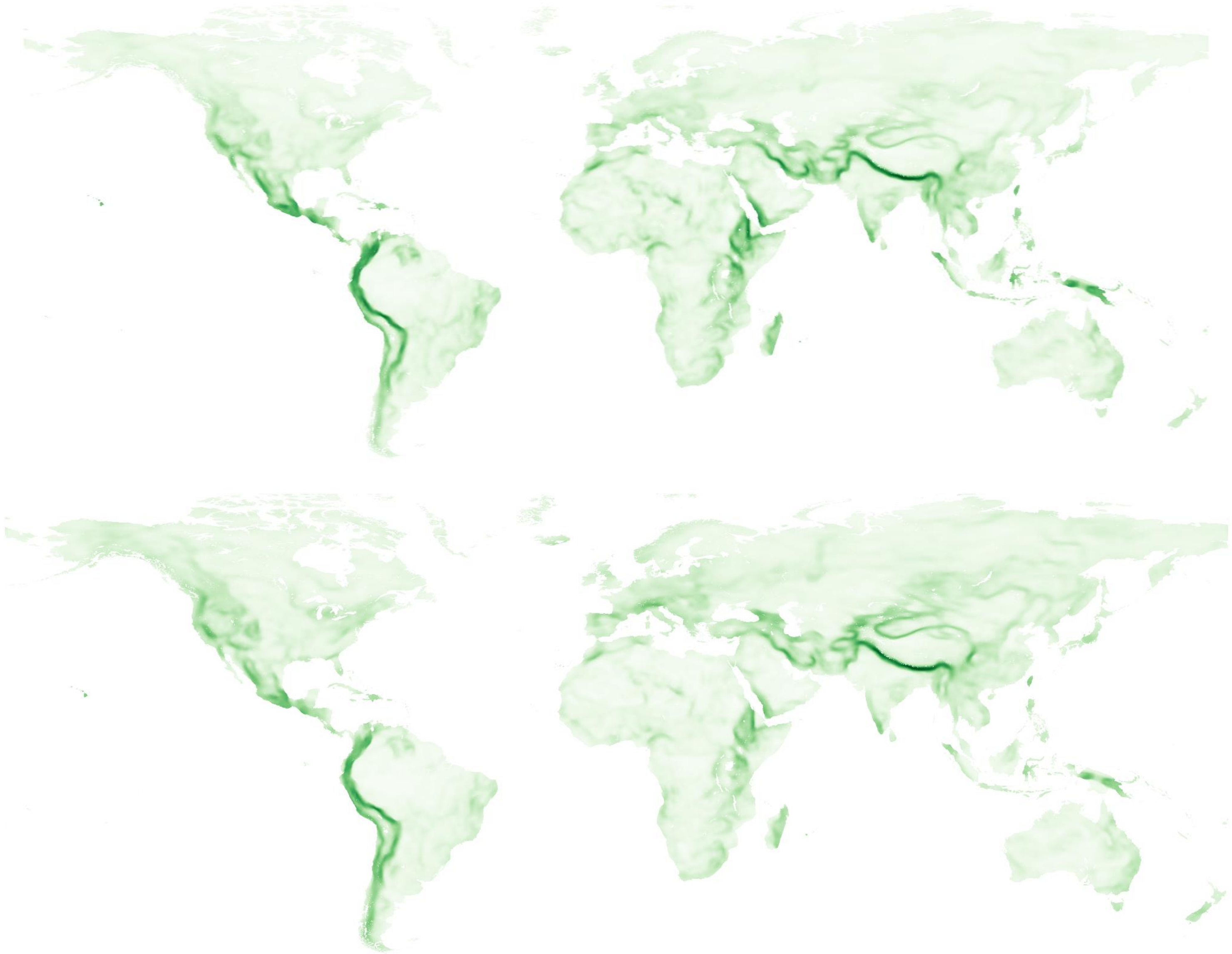

Fig. S21. Global distribution of the expected (A) local turnover and (B) local phylogenetic turnover of fungal communities. D'_{TAX} and D'_{PD} averaged within a 150-km radius. Darker values denote higher turnover.

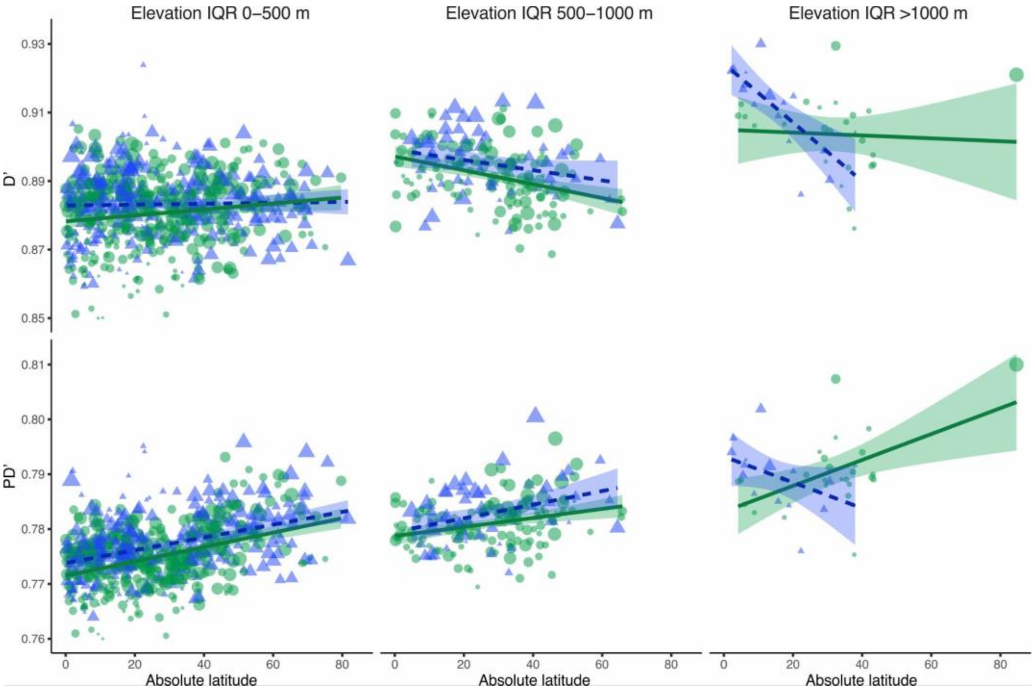

Elevation IQR	Beta (D')	p (D')	Beta (PD')	p (PD')
0-500 m	0.01	0.0007	0.02	<<0.0001
500-1000 m	-0.02	0.0032	0.02	0.0008
>1000 m	0.00	0.7475	0.04	0.0107

Fig. S22. Latitudinal distribution of median values of fungal community taxonomic (D'_{TAX}) and phylogenetic (D'_{PD}) dissimilarities in the ecoregions with different degrees of altitudinal heterogeneity in northern (green circles, solid lines) and southern (blue triangles, dashed lines) hemispheres.

Standard error bounds around regression lines are shown with shaded areas. Altitudinal heterogeneity is defined as the interquartile range (IQR) of elevations within an ecoregion; point size is proportional to the elevation IQR within each IQR class. The table contains standardized regression coefficients and corresponding *P*-values for the effect of latitude revealed by the linear regression model.

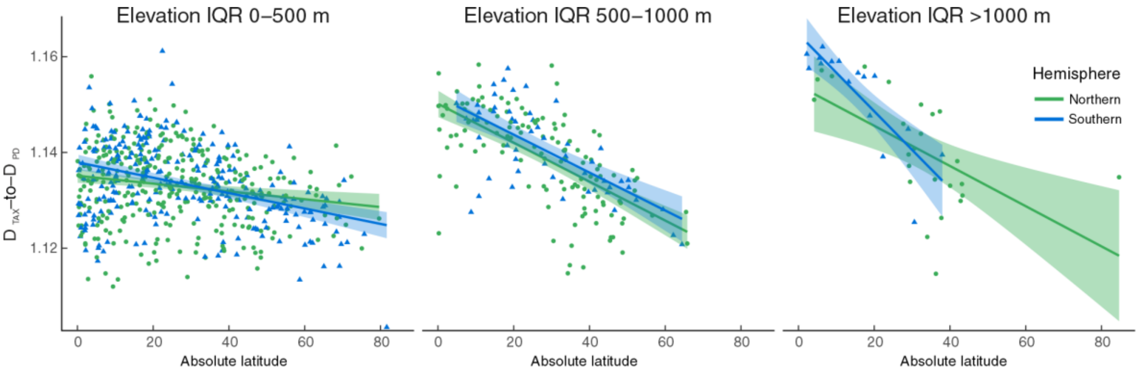

Fig. S23. Latitudinal distribution of ecoregional D'_{TAX}-to-D'_{PD} ratio in ecoregions classified by low, medium, and high altitudinal differences.

Linear regression fits are indicated by lines, and shaded areas represent confidence limits.

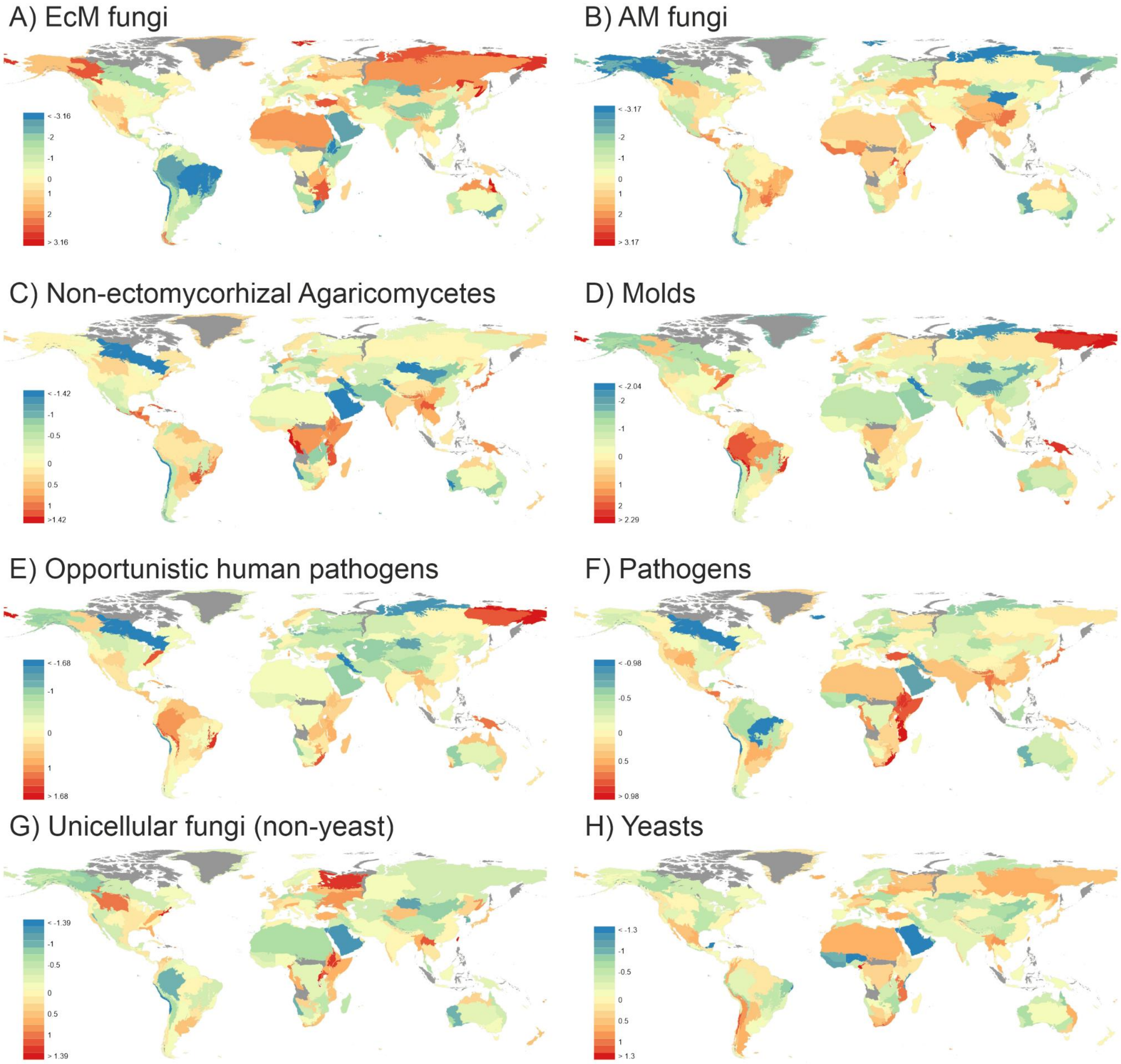

Fig. S24. Gamma diversity of fungal ecological groups at the ecoregion scale.

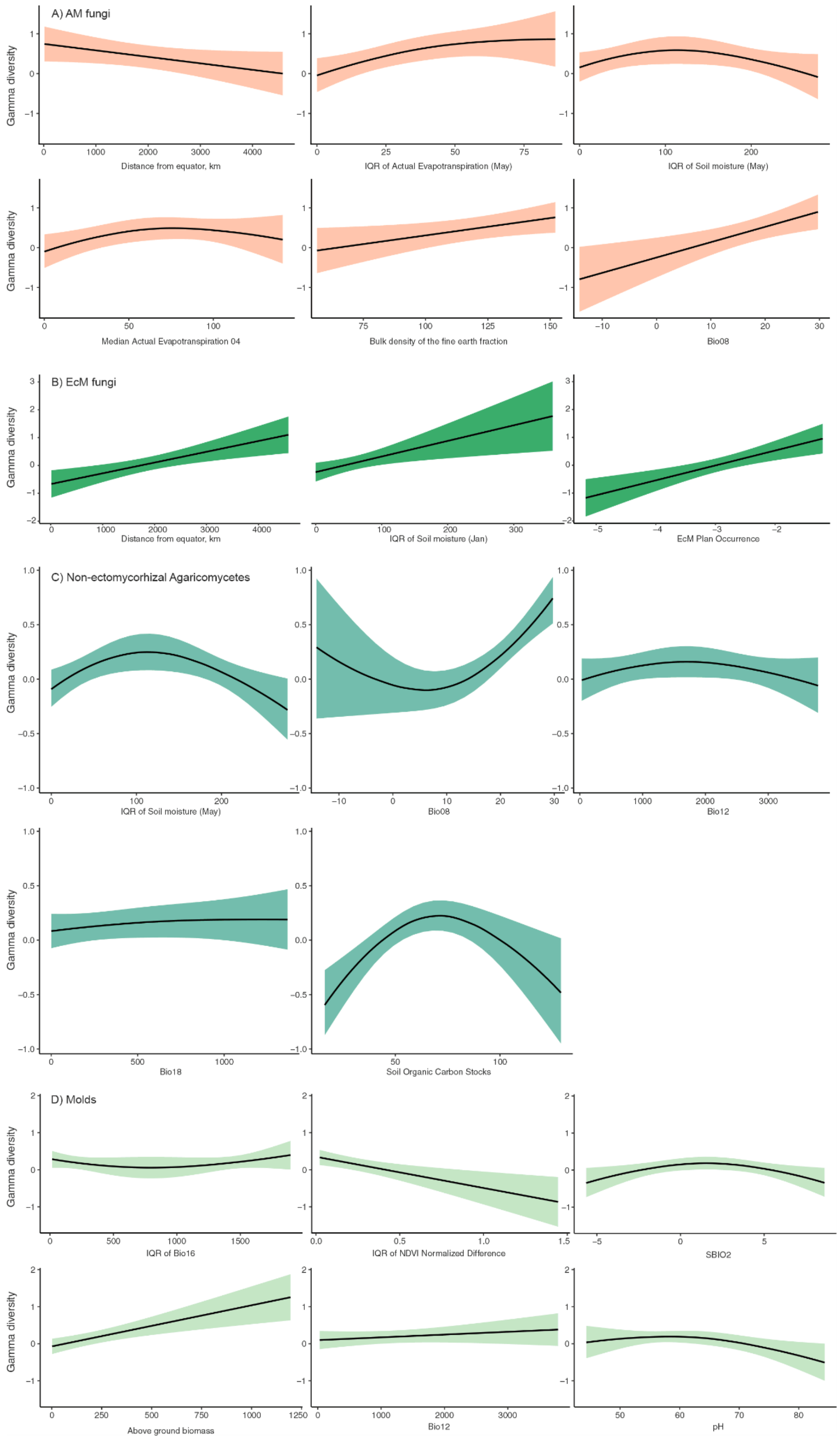

Fig. S25. Marginal effects of the most important environmental predictors on the gamma diversity of fungal ecological groups.

Shaded areas denote the 95% confidence interval on the fitted values.

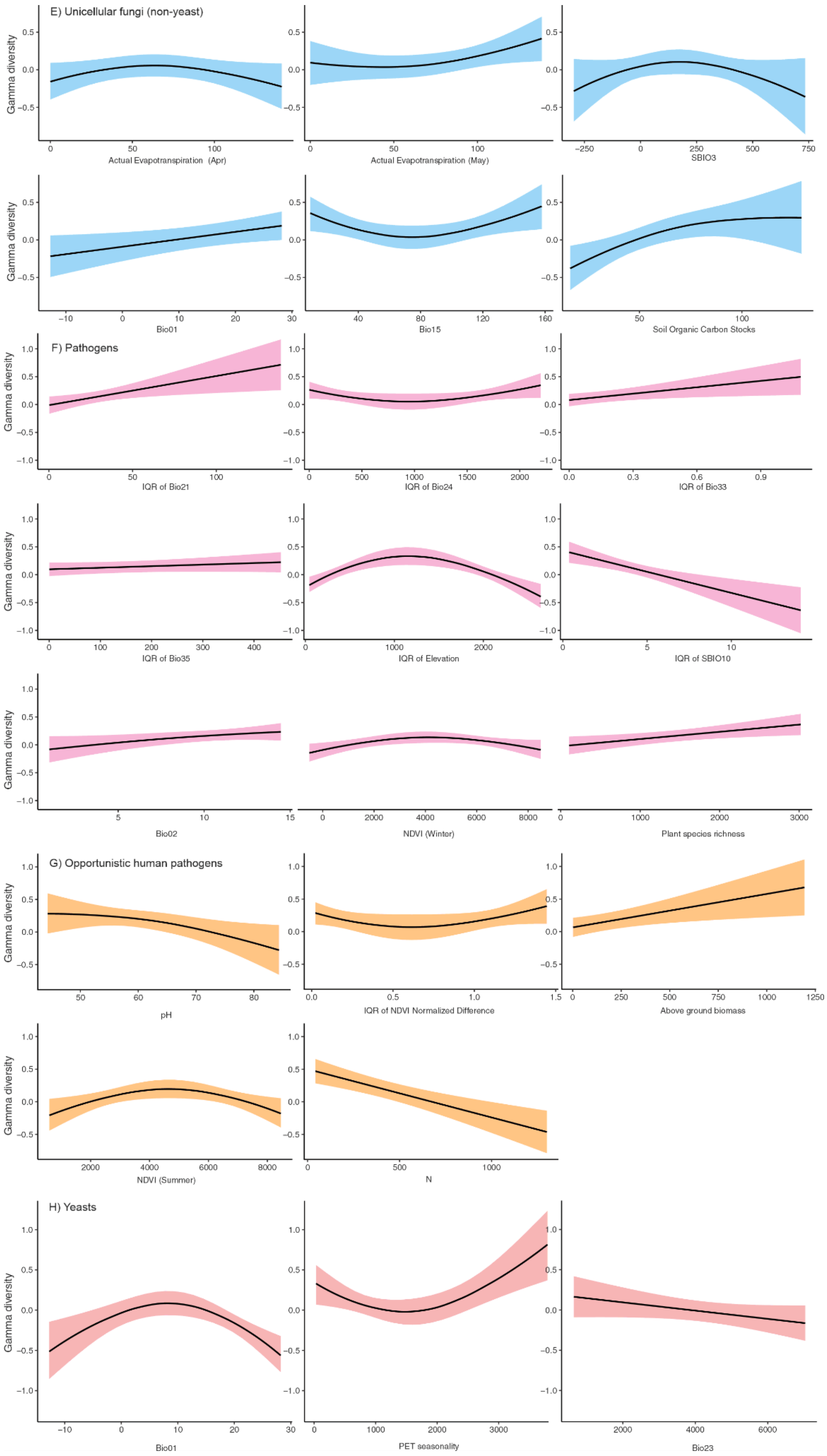

Fig. S25. Marginal effects of the most important environmental predictors on the gamma diversity of fungal ecological groups.

Figures Pertaining to Methodological Approaches

For alpha diversity analyses, we initially compared several metrics, viz. residuals of logarithmically-transformed fungal richness against the logarithm of sequencing depth, residuals from untransformed richness against square-root-transformed and log-transformed sequencing depth (*5*), exclusion of singletons, Shannon index of diversity, traditional rarefaction to minimum common sequencing depth (500 reads), and SRS normalization (*69*) to 500 or 3894 (median) reads (fig. S26). Because the approach including singletons and log-log transformation for selecting residuals resulted in best-supported models (fig. S27), we chose this approach for further analyses.

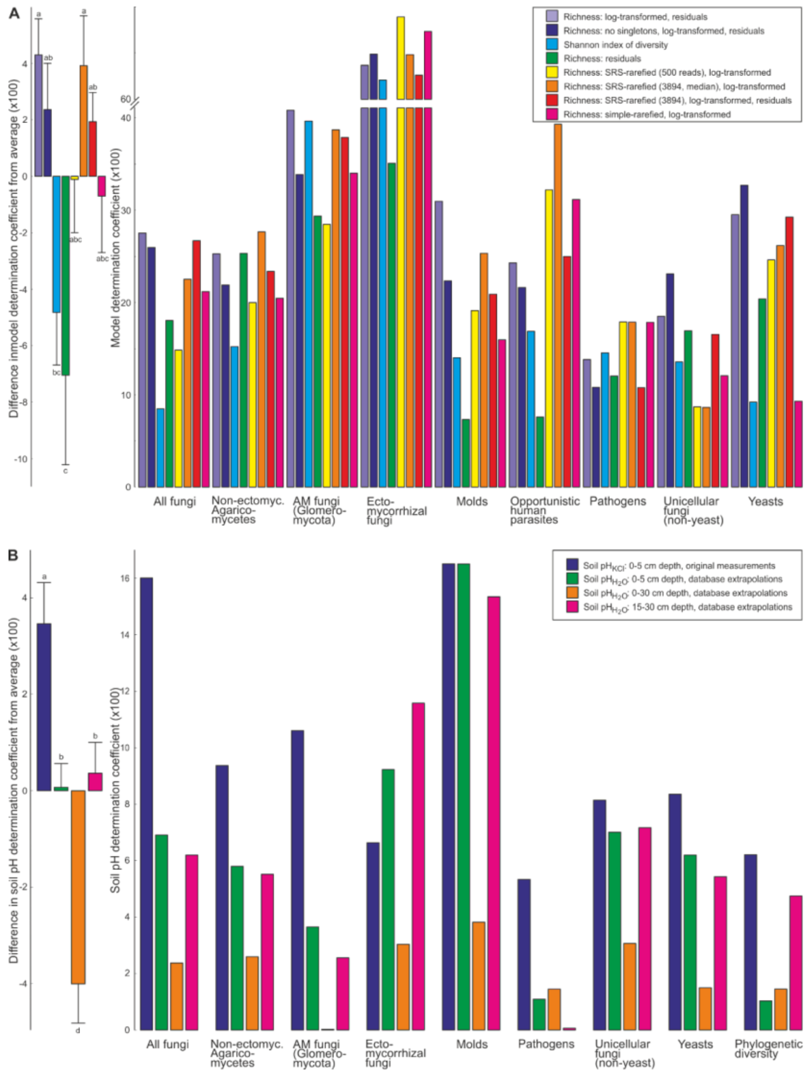

Fig. S26. Standardization effect on richness of fungal communities.

Comparison of (**A**) richness proxies (use of log-transformation, residuals of sequencing depth, SRS or simple rarefaction) and (**B**) measures of soil pH on analytical performance. Relative goodness was estimated based on the determination coefficients of the best models (**A**) or pH-only models (**B**). In the panels to the left, significant among-group differences are indicated with different letters based on Tukey *post hoc* tests; bars, means; whiskers, SE. Soil pH_{KCl} was determined experimentally, whereas pH_{H2O} was obtained from (*61*).

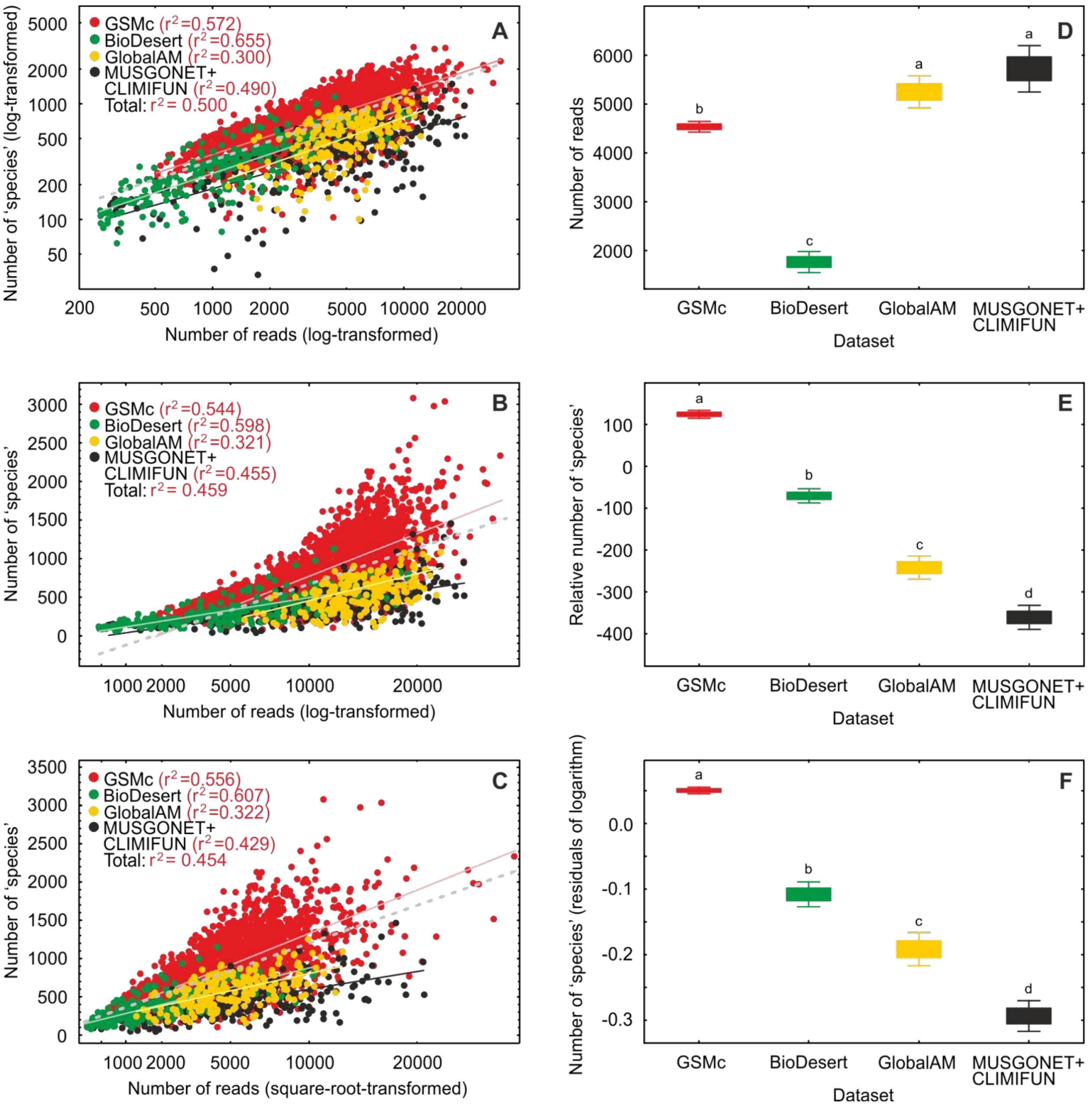

Fig. S27. Relative 'species' accumulation curves (A-C), sequencing depth (D) and 'species' richness (E-F) across four datasets.

(**A**) The log-log relationship between the number of reads and 'species' richness that was used for calculation of residuals and further analyses; (**B-C**) Relatively lower performance of log-linear relationships of log-transformed and square-root-transformed sequencing depth; (**D**) Initial differences in sequencing depth among datasets; (**E-F**) Fungal 'species' richness differences relative to the average in the raw data (**F**) and residuals of the log-log regression analysis (**F**). In **D-F**, boxes indicate standard errors around the mean and whiskers indicate 95% confidence intervals; letters above whiskers indicate statistically significant differences among datasets (using log-transformed data for **D-E**). These analyses indicate that the log-log transformation for calculating residuals is relatively more robust compared to other methods and that richness estimates from studies with different methods cannot be directly compared.

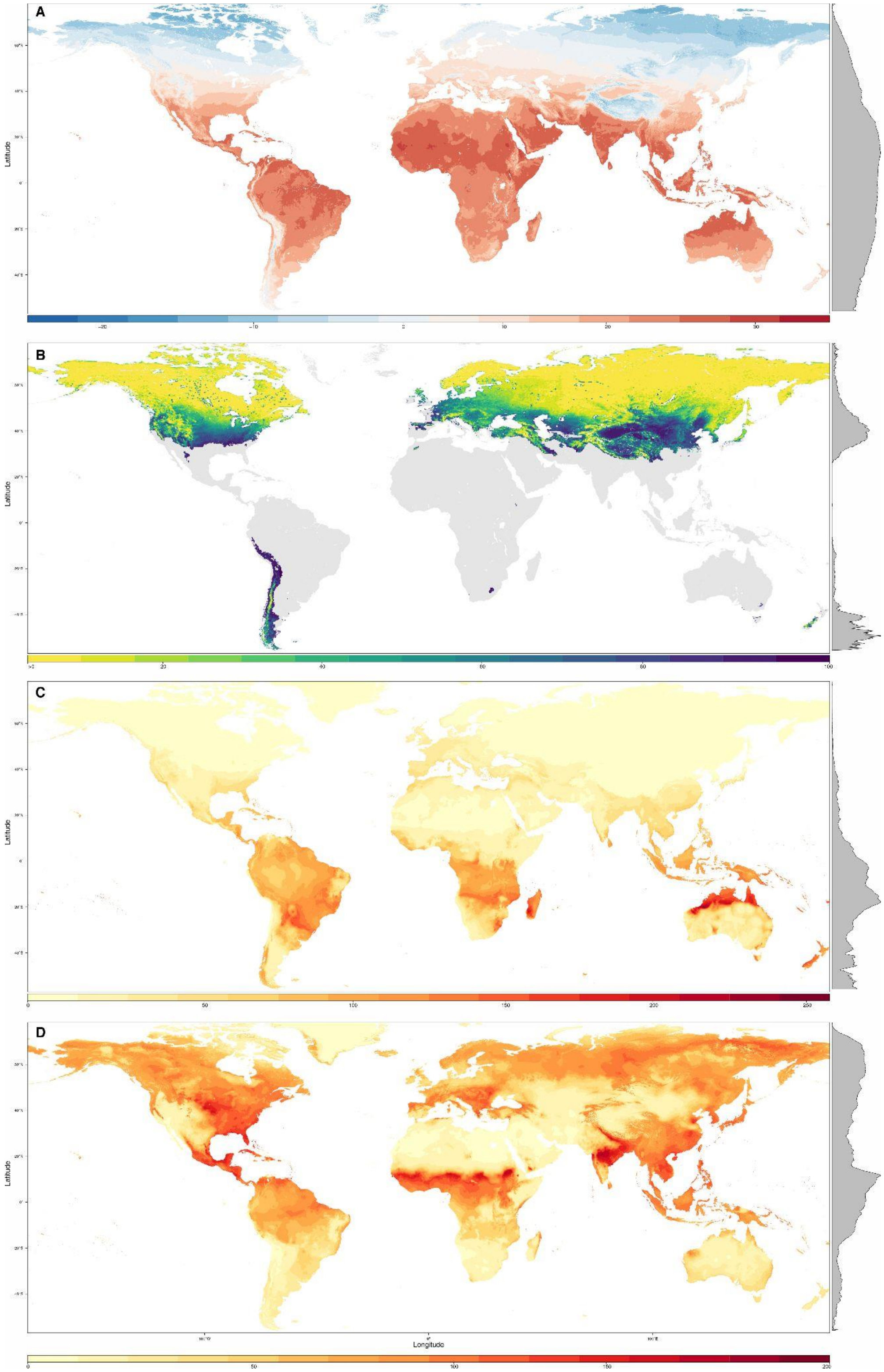

Fig. S28. Maps of the spatial distribution of the environmental variables, associated with fungal richness.

(**A**) annual mean temperature (*56*); (**B**) percent of days of frozen ground without snow (lack of subnivium); (*72*)); and actual evapotranspiration in (**C**) January and (**D**) June (*60*). Marginal distributions of the predictors' values are shown as density plots to the right of the maps.

Supplementary Tables

Table S1. Partitioning of OTUs among fungal ecological groups

Table S2. A list of explanatory variables included in the modeling

Table S3. Fungal richness (S'_{TOT}, consensus map) across different ecoregions and land cover types

Table S4. Importance of key environmental variables in relation to fungal richness and phylogenetic diversity

Table S5. Global variability of predicted local richness of fungal ecological groups

Table S6. Correlations of fungal phylogenetic diversity (S_{PD}) and dispersion (SES_{PD}) with richness of ecological groups (S). LCI and UCI, lower and upper 95% confidence intervals.

Table S7. Divergence of all terrestrial biomes in mycobiota composition (results of PERMANOVA)

Table S8. Average alpha, beta, and gamma diversity of soil fungal communities in different ecoregions (*46)*

Table S9. Impact of geographical factors including area type (continent or island), island area, distance from mainland, and altitudinal span on gamma diversity estimates of soil fungi

Table S10. Correlation matrix of alpha (S') and gamma (G) diversity estimates for soil fungi

Table S11. Key predictors influencing fungal gamma diversity

Table S12. The structure of the datasets used in the analysis (the number of samples and OTUs)

Table S13. Summary of quality-filtered sequencing reads per sample across different datasets

Tables S1–S13 are available online as a Separate .XLSX file under the Supporting Materials for this article.
